# Using structural equation modeling to detect response shifts and true change in discrete variables: an application to the items of the SF-36

**DOI:** 10.1007/s11136-015-1195-0

**Published:** 2015-12-22

**Authors:** Mathilde G. E. Verdam, Frans J. Oort, Mirjam A. G. Sprangers

**Affiliations:** Department of Medical Psychology, Academic Medical Centre, University of Amsterdam, Amsterdam, The Netherlands; Department of Child Development and Education, University of Amsterdam, Postbus 15776, 1001 NG Amsterdam, The Netherlands

**Keywords:** Health-related quality of life (HRQL), Response shift, Structural equation modeling (SEM), Discrete data, Item-level analyses, SF-36 health survey

## Abstract

**Purpose:**

The structural equation modeling (SEM) approach for detection of response shift (Oort in Qual Life Res 14:587–598, 2005. doi:10.1007/s11136-004-0830-y) is especially suited for continuous data, e.g., questionnaire scales. The present objective is to explain how the SEM approach can be applied to discrete data and to illustrate response shift detection in items measuring health-related quality of life (HRQL) of cancer patients.

**Methods:**

The SEM approach for discrete data includes two stages: (1) establishing a model of underlying continuous variables that represent the observed discrete variables, (2) using these underlying continuous variables to establish a common factor model for the detection of response shift and to assess true change. The proposed SEM approach was illustrated with data of 485 cancer patients whose HRQL was measured with the SF-36, before and after start of antineoplastic treatment.

**Results:**

Response shift effects were detected in items of the subscales mental health, physical functioning, role limitations due to physical health, and bodily pain. Recalibration response shifts indicated that patients experienced relatively fewer limitations with “bathing or dressing yourself” (effect size *d* = 0.51) and less “nervousness” (*d* = 0.30), but more “pain” (*d* = −0.23) and less “happiness” (*d* = −0.16) after antineoplastic treatment as compared to the other symptoms of the same subscale. Overall, patients’ mental health improved, while their physical health, vitality, and social functioning deteriorated. No change was found for the other subscales of the SF-36.

**Conclusion:**

The proposed SEM approach to discrete data enables response shift detection at the item level. This will lead to a better understanding of the response shift phenomena at the item level and therefore enhances interpretation of change in the area of HRQL.

**Electronic supplementary material:**

The online version of this article (doi:10.1007/s11136-015-1195-0) contains supplementary material, which is available to authorized users.

## Introduction

Assessment of change in health-related quality of life (HRQL) is important for determining the clinical effectiveness of treatment, as well as for monitoring well-being of individual patients over time. However, comparison of HRQL-scores across time may be invalidated by the occurrence of “response shift”. Response shift refers to a change in respondents’ frames of reference that hinders a meaningful comparison of questionnaire-scores across time. Three different types of response shift are distinguished: recalibration, reprioritization and reconceptualization [38].

Several methodological approaches have been developed for the detection of response shift in HRQL outcomes [37], among which are statistical approaches such as structural equation modeling (SEM) [33]. Advantages of the SEM approach are that it allows for the operationalization of all three types of response shift and that possible response shift effects can be taken into account to assess “true” change. Within the SEM framework, the observed scores (e.g., questionnaire scales) are modeled to be reflective of an underlying unobserved latent variable or common factor (e.g., HRQL). The means and covariances of the observed variables (*y*) are then given by:

1 $${\text{Mean}}(y) = {\varvec{\upmu}} = {\varvec{\uptau}} + {\varvec{\Lambda}}\,{\varvec{\upkappa}},$$

and

2 $${\text{Cov}}\left( {y,y^{\prime } } \right) = {\varvec{\Sigma}} = {\varvec{\Lambda}}\,{\varvec{\Phi}}\,{\varvec{\Lambda}}^{\prime } + {\varvec{\Theta}},$$

where **τ** is a vector of intercepts, **Λ** is a matrix of common factor loadings, **κ** is a vector of common factor means, **Φ** is a matrix containing the variances and covariances of the common factors, ${\varvec{\Lambda}}^{{\prime }}$ denotes the transpose of **Λ**, and **Θ** is a matrix containing the variances and covariances of the residual factors. When SEM is applied to longitudinal data, response shift can be operationalized using SEM parameter estimates, where changes in the pattern of factor loadings (i.e., the pattern of **Λ** indicates which of the factor loadings are free to be estimated) are indicative of reconceptualization, changes in the values of factor loadings are indicative of reprioritization, and changes in intercepts and residual variances are indicative of uniform and nonuniform recalibration, respectively, (see [33] for more details).

The SEM method is especially suited to detect response shift and assess true change in continuous data. The objective of the present paper is twofold. First, we will explain how to analyze discrete data, e.g., ordinal item responses, using the SEM approach. We will show that the model of Eqs. (1) and (2) can still be used, but that the SEM approach needs to be extended to include a modeling stage in which the observed discrete ordinal variables are modeled to be reflective of underlying continuous variables (Stage 1). Stage 1 yields estimates of means and variances and covariances that can be used for the detection of response shift and assessment of true change in Stage 2. Second, we will apply the proposed SEM approach to the discrete ordinal item responses of the SF-36 questionnaire [40] that were obtained from 485 cancer patients, before and after start of antineoplastic treatment.

## SEM approach for discrete data

**Fig. 1:**
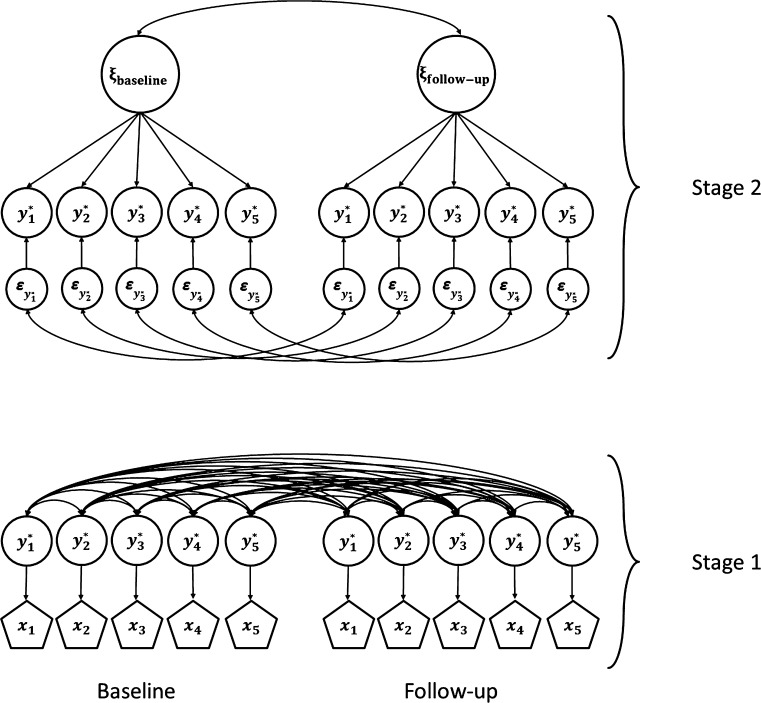
The models of Stage 1 and Stage 2 of the SEM approach for discrete ordinal data. The *pentagons at the bottom* represent observed discrete ordinal variables *x*_1_–*x*_5_, the *circles* with $y_{1}^{*}$–$y_{5}^{*}$ represent the corresponding underlying continuous variables. The same $y^{*}$ feature in Stage 2 (*top* of the figure), as the reflective indicator variables (the *circles* reflect the fact that they are not directly observed). Each y* is associated with a residual factor $\varepsilon$. The residual factors represent everything that is specific to the corresponding y*. Residual factors of the same variable are correlated across measurement occasion. The *circles at the top* are the underlying common factors (*ξ*) at each measurement occasion and represent everything that $y_{1}^{*}$–$y_{5}^{*}$ have in common (e.g., health-related quality of life). In Stage 1, each observed discrete variable *x* is modeled to be reflective of a single underlying continuous variable y*. Assuming a bivariate normal distribution for each pair of y* variables, we can estimate the means $({\varvec{\upmu}}_{{y^{*} }} )$ and variances and covariances $({\varvec{\Sigma}}_{{y^{*} }} )$ on the basis of observed frequencies in the two-dimensional frequency tables of each pair of *x* variables. In Stage 2, the means and variances and covariances of y* are modeled using a common factor model with common factors *ξ*. Across occasion differences in estimates of measurement parameters are indicative of response shift. Specifically, in Stage 1 we investigate invariance of thresholds, and in Stage 2 we investigate invariance of intercepts, factor loadings, and residual variances (see also Table 1)

One of the underlying assumptions of SEM with maximum likelihood (ML) estimation is that the scores of the observed variables follow a multivariate normal distribution. In the case of discrete variables, this assumption is not met, as the responses are limited to a small number of values (e.g., two, three or four response categories). To enable analysis of discrete data, we need to assume that the observed ordinal variables are representations of continuous underlying variables, where lower categories of the observed ordinal variable are related to lower scores on the continuous underlying variable, and vice versa. The model of continuous underlying variables (y*) yields estimates of means $({\varvec{\upmu}}_{{y^{*} }} )$ and variances and covariances $({\varvec{\Sigma}}_{{y^{*} }} ),$ which can be used in subsequent SEM analyses. SEM with discrete data has been explained elsewhere (e.g., [10, 18, 19, 24–27, 32]). Table 1 gives an overview of the SEM approach for discrete data that is used in the present paper, including short descriptions of each step of the approach, the statistical procedures, and the item- and scale characteristics that are required to perform the associated statistical analyses. The steps in Stage 1 and Stage 2 of the SEM approach are similar, but in Stage 1 we operate under the assumption of multivariate normality and investigate the relation of observed scores with single underlying variables, and in Stage 2 we operate under the common factor model and investigate the relation with underlying common factors. Figure 1 shows the Stage 1 and Stage 2 models for an example of five observed discrete ordinal variables measured at two occasions.

**Table 1:** Stage 1 and Stage 2 of the SEM approach for discrete data

Stage 1	Measurement model: observed discrete ordinal scores *x* are representations of underlying, continuous scores y*		
	What	How	Requirements
Step 1	Test the assumption of underlying, bivariate normally distributed continuous scores for each pair of discrete ordinal variables^a^	The likelihood ratio (LR) test statistic can be used to test the hypothesis of underlying bivariate normal distributed continuous variables. The LR test is a test of exact fit^b^, the root-mean-square error of approximation (RMSEA) can be used to evaluate approximate fit, with the criterion that RMSEA values should not be larger than 0.1 [21]	Applicable only with three or more response categories^c^
Step 2	Test the assumption of invariance of thresholds across occasions for each discrete ordinal variable^d^	The difference in LR test statistics can be used to test the difference in exact fit [21]. The expected cross validation index (ECVI; [6]) can be used to test the difference in approximate fit, where a value that is significantly larger than zero indicates that the more restricted model (i.e., the model with equality constraints on the thresholds) has significantly worse approximate fit	Applicable only with 4 or more response categories^e^
Step 3	Investigate recalibration response shift as indicated by non-invariance of thresholds across occasions in the Stage 1 measurement model	To investigate whether the non-invariance of thresholds can be attributed to specific threshold parameters, the tenability of the equality restrictions across measurement occasions can be evaluated further. For example, by testing the invariance of individual thresholds. The LR test statistics can be used to test the difference in exact fit, and the ECVI difference can be used to test the difference in approximate fit	Applicable only with four or more response categories^f^
Step 4	Assess differences in estimated means of the underlying variables (i.e., true change) across measurement occasions	The effect size can be estimated by *d* = $\frac{{\hat{\mu }_{2} - \hat{\mu }_{1} }}{{\hat{\sigma }_{\text{diff}} }},$ where $\hat{\mu }_{1}$ and $\hat{\mu }_{2}$ are the estimated means of the underlying variables y* at occasions 1 and 2, and $\hat{\sigma }_{\text{diff}}$ is given by $\sqrt {\hat{\sigma }_{j1,j1}^{2} + \hat{\sigma }_{j2,j2}^{2} - 2\hat{\sigma }_{j2,j1} }$), where variances $\hat{\sigma }_{j1,j1}^{2}$ and $\hat{\sigma }_{j2,j2}^{2} ,$ and covariance $\hat{\sigma }_{j2,j1}$ are elements from the estimated covariance matrix ${\hat{\mathbf{\varSigma }}}_{{y^{*} }} ,$ as implied by the final model from Step 2	Applicable only with two or more response categories

Stage 2	Measurement model: continuous scores y* are explained by a common factor model		
	What	How	Application
Step 1	Test the common factor model by fitting it to the means, variances, and covariances of continuous scores y* obtained in Stage 1	The Chi-square test can be used to evaluate exact goodness-of-fit, where a significant Chi-square indicates a significant difference between data and model. The RMSEA value can be used as a measure of approximate goodness-of-fit, where values below .08 indicate “reasonable” approximate fit and below .05 “close” approximate fit [7]. The hypothesis of close fit can be evaluated using the 90 % confidence intervals of the RMSEA value	Applicable only with three or more variables^g^
Step 2	Test the assumption of invariance of measurement parameters associated with response shift across measurement occasions	The Chi-square difference test can be used to test the difference in exact fit, where a significant Chi-square difference indicates that the no response shift model (with invariance restrictions imposed) has significantly worse fit as compared to the measurement model (without invariance restrictions). The ECVI difference can be used to test equivalence in approximate model fit	Applicable only with two or more variables^h^
Step 3	Investigate recalibration, reprioritization, and reconceptualization response shift as indicated by non-invariance of intercepts, factor loading values, and factor loading patterns across occasions in the Stage 2 measurement model	Improvement in model fit for each modification can be tested using the Chi-square difference test to evaluate differences in exact fit and the ECVI difference test to evaluate differences in approximate fit. In addition, the final model can be compared to the measurement model to test equivalence of exact and approximate fit	Applicable only with two or more variables^i^
Step 4	Assess differences in estimated means of the common factors (i.e., true change) across measurement occasions Decompose change in the means of the continuous variables y* across occasions into true change, recalibration response shift, and reprioritization or reconceptualization response shift^j^	The effect size of true change in the common factors between occasion 1 and 2 can be estimated by $d \, = \frac{{\hat{\mu }_{2} - \hat{\mu }_{1} }}{{\hat{\sigma }_{\text{diff}} }},$ where $\hat{\sigma }_{\text{diff}}$ is given by $\sqrt {\hat{\varphi }_{r1,r1}^{2} + \hat{\varphi }_{r2,r2}^{2} - 2\hat{\varphi }_{r2,r1} } .$ The variances $\hat{\varphi }_{r1,r1}^{2}$ and $\hat{\varphi }_{r2,r2}^{2} ,$ and covariance $\hat{\varphi }_{r2,r1}$ are elements from the estimated covariance matrix ${\hat{\mathbf{\varPhi }}}$ of the final model from Step 3 Change in the means of the observed variables can be decomposed as follows: $\mu_{2} - \mu_{1} = (\tau_{2} - \tau_{1} ) + ((\varLambda_{2} - \varLambda_{1} )\kappa_{2} ) + \varLambda_{1} \kappa_{2} .$ Subsequently, effect sizes for modeled change $(\mu_{2} - \mu_{1} ),$ recalibration $(\tau_{2} - \tau_{1} ),$ reprioritization and reconceptualization $((\varLambda_{2} - \varLambda_{1} )\kappa_{2} )$ and true change $(\varLambda_{1} \kappa_{2} )$ can be calculated using the standard deviation of change $\hat{\sigma }_{\text{diff}}$ (as in Step 4 of Stage 1)	Applicable only with two or more variables

^a^That is, 2*n* ^2^ − *n* tests for 2*n* ^2^ − *n* pairs of 2*n* variables

^b^To guard against inflation of family wise Type I error, a Bonferroni-corrected significance level can be used to take into account multiple comparisons, where α* = *α*/(2*n* ^2^ − *n*)

^c^When there are only two response categories, there is not enough information to evaluate the LR test statistic for pairs of items. One can instead test the assumption of underlying, trivariate normally distributed continuous scores for each triplet of dichotomous variables

^d^That is, *n* tests for 2*n* variables

^e^When there are only two or three response categories, there is not enough information to evaluate the difference in LR test statistic

^f^When there are only two, three or four response categories, it is not possible to attribute possible non-invariance to a specific threshold

^g^When there are only two variables, then we need additional restrictions on model parameters (e.g., equality restriction on factor loadings or restricting the residual covariances to zero) to achieve identification

^h^When the variables have only two response categories then we cannot test the invariance of factor loadings (see Supplement 1.4)

^i^When there are only two variables, it is possible to test the invariance of intercepts but, if significant, it is not possible to identify which of the two variables has response shift

^j^“True” change is represented by change in common factor means, recalibration is represented by change in the intercepts, and reprioritization and reconceptualization are represented by change in the factor loadings

### Stage 1: Observed discrete ordinal scores *x* are representations of underlying, continuous scores y*

Suppose we have an ordinal variable *x* with categories labeled 1, 2, and 3. The relations between the observed categories of the ordinal variable and the underlying continuous variable (y*) are defined using thresholds (*δ*), where:

3 $$\begin{aligned} & x = 1\quad {\text{if}}\,y^{*} < \delta_{1} , \\ & x = 2\quad {\text{if}}\,\delta_{1} < y^{*} < \delta_{2} , \\ & x = 3\quad {\text{if}}\,y^{*} > \delta_{2} . \\ \end{aligned}$$

In general, with *m* categories:

4 $$x = i\quad {\text{if}}\,\delta_{i - 1} < y^{*} < \delta_{i} ,$$

where

$$\delta_{0} \to - \infty ,$$

and

$$\delta_{m} \to + \infty .$$

**Fig. 2:**
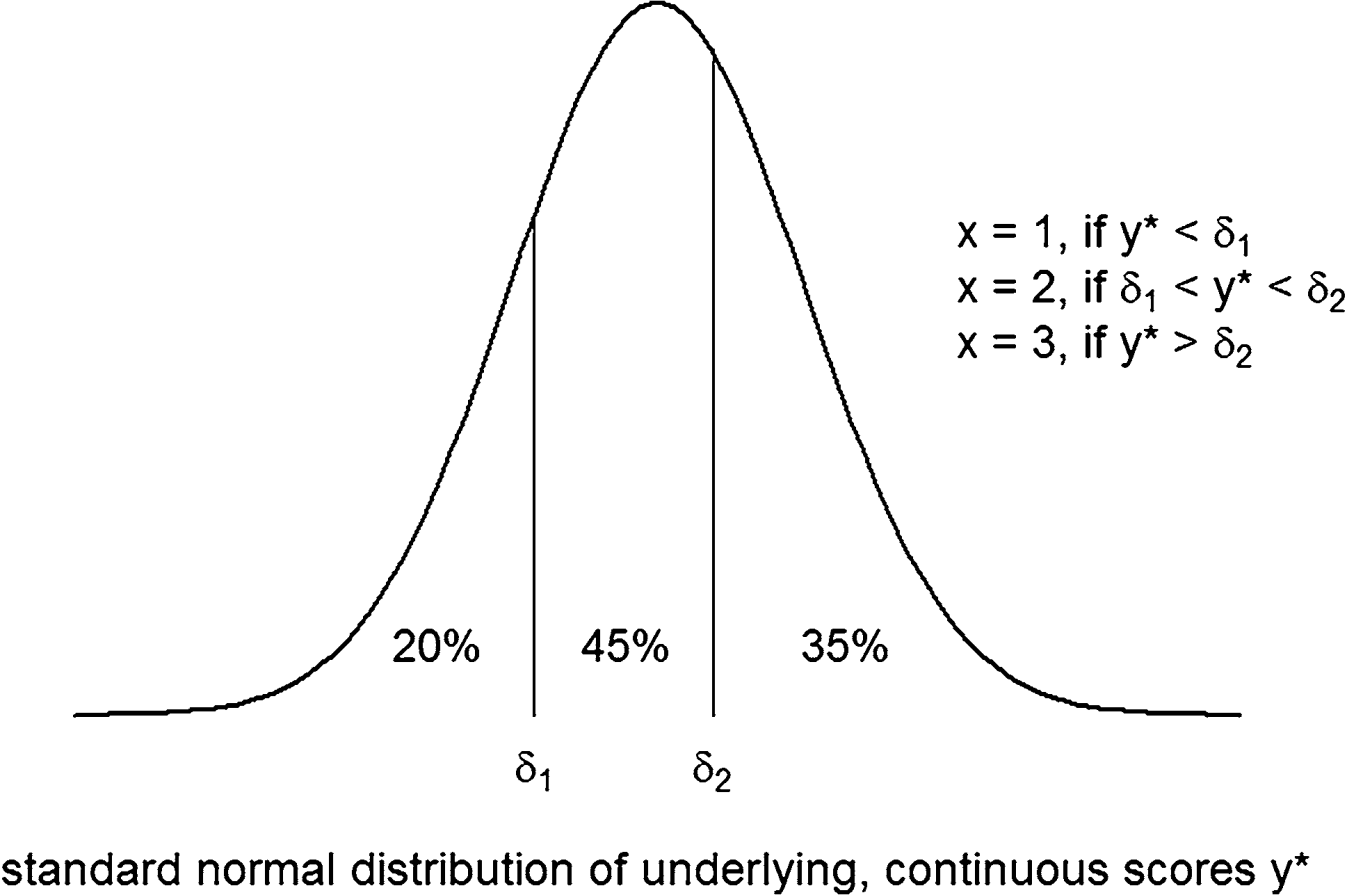
The estimation of thresholds (*δ*): observed discrete scores *x* are representations of underlying continuous scores y*. There are 20, 45 and 35 % observed responses in categories 1, 2 and 3, respectively. The first threshold is located where the area under the curve to the left of the threshold is 20 % (*δ*_1_ = −0.842). The second threshold is located where the area under the curve to the left of the threshold is 65 % (*δ*_1_ = 0.385)

The number of thresholds is thus equal to the number of response categories minus one. When we assume the underlying variable to follow a standard normal distribution (i.e., with a mean of zero and variance of one), then the threshold *δ*_*i*_ defines an area under the curve left from the threshold that is equal to the proportion of observed responses in category *i* or lower (see Fig. 2).

The correlations between the underlying variables can be estimated by assuming bivariate standard normal distributions. With two ordinal variables *x*_1_ and *x*_2_, the sample observations can be represented by a contingency table that contains the number of responses (*n*_*ij*_) of category *i* on variable *x*_1_ and category *j* on variable *x*_2_. When we assume bivariate normality, we can estimate thresholds and correlations that yield expected frequencies that are as close as possible to the observed frequencies (see [21] for more details). When both variables have more than two response categories, the correlation is called a “polychoric” correlation; when both variables have only two response categories, it is called a “tetrachoric” correlation. These correlations indicate what the Pearson correlation would have been if these variables had been measured on a continuous scale.

#### Step 1: Testing the underlying bivariate normality

Polychoric correlations are estimated under the assumption of bivariate normality of the underlying continuous variables. The tenability of this assumption can be evaluated by comparing the expected proportions under bivariate normality to the observed sample proportions (see Table 1 for details on evaluation of model fit). When the hypothesis of bivariate normality holds for all pairs of variables, the assumption of multivariate normality is also supported. If the hypothesis of bivariate normality does not hold, then this indicates that the assumption of multivariate normality is not tenable. A possible solution for this problem is to eliminate the offending variable(s).

#### Step 2: Testing invariance of thresholds across measurement occasions

When the same variables are measured repeatedly (i.e., in longitudinal assessment), the imposition of invariant thresholds across measurement occasions is required for a common scale (see Supplement 1.1 for more details). The tenability of this restriction can be tested for each pair of variables by comparing the model with equality constraints on the thresholds to the Step 1 model without equality constraints on the thresholds (see Table 1). When the difference in model fit is significant, the hypothesis of equal thresholds across measurements must be rejected.

#### Step 3: Investigating possible non-invariance of thresholds

When the assumption of invariant thresholds across measurement occasions does not hold, this can be taken as an indication of recalibration response shift. Differences in thresholds of the same variable across measurement occasions indicate that the association between the scores of the underlying variable and the observed response category of that variable has changed; the underlying variables are not measured on the same scale. Occurrence of recalibration response shift in Stage 1 can be taken into account by allowing threshold parameters to be freely estimated across measurement occasions.

We introduce the term recalibration response shift in Stage 1, but want to emphasize that it is different from recalibration response shift in Stage 2. In Stage 1, differences between thresholds are detected given the model of bivariate normality of single underlying variables, and thus recalibration response shift is defined relative to the scale of the underlying variable. In Stage 2, differences between intercepts are detected given the common factor model and thus recalibration response shift is defined relative to the scale of the common factor (e.g., HRQL), and thus relative to the other variables measuring the same common factor.

To further investigate recalibration response shift, the tenability of equality restrictions on thresholds across measurement occasions can be evaluated for each threshold separately (see Table 1). This could give an indication as to whether the changes in the association between the scores of the underlying variable and the observed response categories can be attributed to a specific part of the measurement scale (e.g., non-invariance of the first threshold parameter would indicate that there is a shift in the meaning of the response scale’s values at the lower end of the measurement scale).

#### Step 4: Assessment of true change

To assess true change in the underlying variables, we can compare estimated means of the model from Step 2 across measurement occasions (see [21], for more details on the estimation of means of the underlying variables under equal thresholds). As invariant thresholds are required to enable a valid comparison of means of the underlying variables, true change can only be assessed for those variables for which the hypothesis of equal thresholds across measurements holds. True change estimates can be compared to observed change (i.e., the mean differences of the observed discrete variables). Table 1 provides information on the calculation of effect size indices of change. Effect size values of 0.2, 0.5, and 0.8 are considered “small,” “medium,” and “large” [12].

In other procedures for discrete data analyses, the tenability of bivariate normality and invariance of thresholds is usually assumed but not evaluated. By using the proposed four steps, we want to show that the underlying assumptions of the model of Stage 1 can be tested (i.e., Steps 1 and 2) that testing these assumptions can have important consequences (i.e., selection of items in Step 1), and may provide interesting information with regard to possible violations of these assumptions (i.e., recalibration response shift in Step 3), which will lead to a more valid interpretation of change (i.e., Step 4).

### Stage 2: Continuous scores y* are explained by a common factor model

${\varvec{\Sigma}}_{{y^{*} }}$ and ${\varvec{\upmu}}_{{y^{*} }}$ can be used in subsequent SEM analyses in the same way as for continuous variables, using the four steps as proposed by Oort [33]. However, the ML estimation method cannot be used with discrete data. One of the alternative estimation methods that can be used to yield unbiased parameter estimates and standard errors, and appropriate goodness-of-fit measures is the “weighted least squares” (WLS; [5]) method (see Supplement 1.2 for more details). When there are only two observed variables (e.g., a scale that consists of only two items), or when the observed variables are dichotomous (i.e., when analyzing a matrix of tetrachoric correlations), the SEM approach requires additional adaptations that are explained in Supplements 1.3 and 1.4, respectively.

#### Step 1: Testing the measurement model

The measurement model is a multidimensional model that includes multiple measurement occasions, but without any across occasion constraints (see Fig. 1 for an example of the measurement model with two measurement occasions). To achieve identification of all model parameters, scales and origins of the common factors can be established by fixing the factor means at zero and the factor variances at one. To test whether the measurement model holds, goodness-of-fit can be assessed using the WLS Chi-square test statistic (see Table 1).

#### Step 2: Testing the invariance of measurement parameters across measurement occasions

In Step 2, a model of no response shift is fitted to the data, where all measurement parameters associated with response shift are constrained to be equal across measurements. To achieve identification of model parameters, only first occasion common factor means and variances are fixed; factor means and variances at successive occasions are then identified due to invariance constraints on intercepts and factor loadings. To test for the presence of response shift, the no response shift model can be compared to the measurement model (see Table 1). If the invariance restrictions of the no response shift model lead to a significant deterioration in model fit, this indicates the presence of response shift.

#### Step 3: Investigating possible response shift effects

In case of response shift, a step-by-step modification of the no response shift model can be used to arrive at the response shift model in which all apparent response shifts are taken into account. Response shift is operationalized as across measurement occasion differences between the pattern of common factor loadings (reconceptualization), values of common factor loadings (reprioritization), differences between intercepts (uniform recalibration), and between residual variances (nonuniform recalibration). The identification of possible response shift effects can be guided by inspection of significant modification indices [20], correlation residuals (>0.10), or by an iterative approach where each constrained parameter associated with response shift is set free to be estimated one at a time, and the freely estimated parameter that leads to the largest improvement in fit is included in the model (see Table 1 for details on model fit evaluation).

#### Step 4: Assessment of true change

The parameter estimates of the final model, the response shift model in which all response shifts have been taken into account, can be used for the assessment of true change in the common factors (see Table 1).

In addition, evaluation of response shifts and true change for each individual variable can be done using the decomposition of change as proposed by Oort [33]. The change that is modeled using the common factor model is decomposed into change due to differences in intercepts (i.e., recalibration), change due to differences in factor loadings (i.e., reconceptualization and reprioritization), and change due to difference in the common factor means (i.e., true change). Table 1 provides information on the calculation of effect size indices of change.

## Application

### Patients

A total of 485 cancer patients undergoing active antineoplastic treatment were recruited in a cancer treatment center in Amsterdam. All patients were starting a new course of chemotherapy or radiotherapy. HRQL was assessed before the start of treatment, approximately 4 weeks after start of treatment, and approximately 4 months after start of treatment (see [1] for more details on data collection). For this study, we will only use the data obtained at baseline (pre-test) and immediate follow-up (post-test at 4 weeks). Attrition rate between the baseline and immediate follow-up period was 7.8 % (*N* = 38).

### Measures

HRQL was assessed with the Dutch language version [1] of the SF-36 health survey [40]. The items of the SF-36 health survey can be clustered into eight subscales: mental health (MH; five items; six response categories), general physical health (GH; five items; five response categories), physical functioning (PF; ten items; three response categories), role limitations due to physical health (RP; four items; two response categories), bodily pain (BP; two items; five and six response categories, respectively), social functioning (SF; two items; five response categories), role limitations due to emotional health (RE; three items; two response categories), and vitality (VT; four items; six response categories). The eight subscales can be grouped into two summary measures: MH (i.e., MH, SF, RE and VT) and physical health (i.e., GH, PF, RP and BP). In addition, there is one item on Health Comparison (HC; one item; five response categories). Item response categories were coded such that higher scores indicate better functioning or better health. Missing item responses (0–1.6 %) were replaced by the nearest integer after expectation–maximization [12]. Imputation was only considered for data of patients who had <8 missing item responses to warrant reliability of imputation results. The total study sample therefore consists of 437 patients. Table 2 contains an overview of background variables and clinical variables of the selected study sample and the group of patients that was excluded due to attrition or due to too many missing values. There were no significant differences between the two groups with regard to age, gender, education, marital status, primary tumor site (breast, colorectal, lung or other), treatment modality (chemotherapy, radiotherapy, or combination therapy), and stage of disease (local or loco-regional vs. metastatic). The selected patients showed a significantly higher Karnofsky performance [22] and relatively fewer progressive tumors as compared to the excluded patients.

**Table 2:** Background and clinical variables of the selected study sample (*N* = 437) and the group of patients that was excluded due to attrition or due to too many missing values (*N* = 49)

Variables	Selected study sample	Excluded sample
	Mean (SD)	Mean (SD)
Age	57.0 (12.1)	60.0 (12.0)
Karnofsky performance*	78.4 (13.7)	74.2 (13.0)

	*N* (%)	*N* (%)
Gender		
Men	179 (41 %)	25 (52 %)
Women	256 (59 %)	23 (48 %)
Education		
Primary school	57 (13 %)	7 (15 %)
Lower secondary school	186 (43 %)	19 (40 %)
Higher secondary school	35 (8 %)	3 (6 %)
MBO	81 (19 %)	8 (17 %)
HBO	45 (10 %)	5 (10 %)
University	29 (7 %)	6 (13 %)
Marital status		
Alone	33 (8 %)	5 (10 %)
Married	331 (77 %)	37 (77 %)
Divorced	30 (7 %)	2 (4 %)
Widowed	38 (9 %)	4 (8 %)
Tumor site		
Breast	158 (36 %)	12 (25 %)
Colorectal	105 (24 %)	12 (25 %)
Lung	130 (30 %)	20 (42 %)
Other	44 (10 %)	4 (8 %)
Treatment modality		
Radiotherapy	220 (50 %)	23 (48 %)
Chemotherapy	203 (47 %)	25 (52 %)
Combination therapy	12 (3 %)	0 (0 %)
Stage of disease		
Local/loco-regional	260 (60 %)	23 (48 %)
Metastatic	171 (40 %)	25 (52 %)
Tumor response*		
Progressive	44 (10 %)	14 (48 %)
Regressive	79 (18 %)	5 (17 %)
No response	311 (72 %)	10 (35 %)

Significant differences between the selected study sample and the excluded sample were evaluated with independent sample *t* tests for continuous variables and Chi-square test statistics for categorical variables

* Differences between the groups were significant at *α* = 0.05

### Procedure

The SEM approach for discrete data was applied to all items of the SF-36. In order to reduce model complexity and facilitate interpretation of results, analyses were done for each subscale of the SF-36 separately. The information provided in the SF-36 manual about the clustering of items and published results of principal components analyses of the SF-36 [40] were used to establish the measurement model of each subscale. Response shift was operationalized as across occasion differences between the values of common factor loadings (reprioritization), and differences between intercepts (uniform recalibration). An iterative procedure was used to investigate possible response shift effects, where the across occasion constraints on the parameters associated with response shift were freed one at a time. The freely estimated parameters that were associated with the largest improvement in model fit were included in the model. Reconceptualization response shift was investigated by checking the significance of factor loading parameters (i.e., an item with an insignificant factor loading is not indicative of the common factor). Reconceptualization response shift due to other factors (e.g., other subscales, demographic or clinical variables) was not investigated. The investigation of differences between residual variances (nonuniform recalibration) is straightforward and does not require adaptations to the response shift detection procedure. As the residual factors do not affect assessment of true change, the residual variances are not considered in the present article. Statistical analyses were performed using the PRELIS (Stage 1) and LISREL (Stage 2) programs [20]. Syntax files for reported analyses are available in appendix A of Electronic Supplementary Material (Stage 1) and appendix B of Electronic Supplementary Material (Stage 2). Appendix C of Electronic Supplementary Material provides syntaxes that were used to calculate approximate fit indices (RMSEA and ECVI) with associated confidence intervals, Chi-square difference tests (CHISQ_diff_), and ECVI difference tests (ECVI_diff_). The data are available upon request from the authors.

### Results

Frequency distributions for the items of the SF-36 that were used for analyses can be found in Table 3. Results of statistical analyses from Steps 1–3 of Stage 1 and Stage 2 are presented in Tables 4 and 5, respectively. Estimates of change from Step 4 of both stages are displayed in Table 6. We report results for each subscale of the SF-36 separately. Results of the subscale MH are reported in detail, so that results of other subscales can be reported more concise.

**Table 3:** Frequency distributions of the items of the SF-36 at baseline and follow-up that were used for statistical analyses (*N* = 437)

Item	Time	Response categories					
		1	2	3	4	5	6
Mental health (MH)							
24 Have you been a very nervous person?	Baseline	14 (3 %)	30 (7 %)	55 (13 %)	182 (42 %)	91 (21 %)	64 (15 %)
	Follow-up	10 (2 %)	16 (4 %)	35 (8 %)	154 (35 %)	118 (27 %)	103 (24 %)
25 Have you felt so down in the dumps that nothing could cheer you up?	Baseline	7 (2 %)	13 (3 %)	24 (6 %)	80 (18 %)	112 (26 %)	200 (6 %)
	Follow-up	2 (0 %)	7 (2 %)	16 (4 %)	76 (17 %)	136 (31 %)	199 (46 %)
26 Have you felt calm and peaceful?	Baseline	23 (5 %)	55 (13 %)	100 (23 %	69 (16 %)	141 (32 %)	48 (11 %)
	Follow-up	20 (5 %)	45 (10 %)	114 (26 %)	45 (10 %)	167 (38 %)	45 (10 %)
28 Have you felt downhearted and blue?	Baseline	8 (2 %)	17 (4 %)	33 (8 %)	145 (33 %)	119 (27 %)	114 (26 %)
	Follow-up	7 (2 %)	12 (3 %)	22 (5 %)	153 (35 %)	120 (28 %)	122 (28 %)
30 Have you been a happy person?	Baseline	20 (5 %)	22 (5 %)	85 (20 %)	48 (11 %)	135 (31 %)	126 (29 %)
	Follow-up	21 (5 %)	29 (7 %)	81 (19 %)	52 (12 %)	154 (35 %)	99 (23 %)
General physical health (GH)							
1 In general, would you say your health is…?	Baseline	50 (12 %)	153 (35 %)	162 (37 %)	40 (9 %)	31 (7 %)	
	Follow-up	32 (7 %)	179 (41 %)	174 (40 %)	40 (9 %)	11 (3 %)	
33 I seem to get sick a little easier than other people	Baseline	24 (6 %)	29 (7 %)	118 (27 %)	60 (14 %)	205 (47 %)	
	Follow-up	20 (4 %)	41 (9 %)	131 (30 %)	59 (14 %)	185 (42 %)	
34 I am as health as anybody I know	Baseline	94 (22 %)	100 (23 %)	102 (23 %)	76 (17 %)	64 (15 %)	
	Follow-up	99 (23 %)	91 (21 %)	125 (29 %)	73 (17 %)	48 (11 %)	
35 I expect my health to get worse	Baseline	46 (11 %)	56 (13 %)	172 (39 %)	58 (13 %)	104 (24 %)	
	Follow-up	35 (8 %)	47 (11 %)	197 (45 %)	56 (13 %)	101 (23 %)	
36 My health is excellent	Baseline	130 (30 %)	71 (16 %)	80 (18 %)	101 (23 %)	54 (12 %)	
	Follow-up	131 (30 %)	87 (20 %)	71 (16 %)	112 (26 %)	35 (8 %)	
Physical functioning (PF)							
3 Vigorous activities	Baseline	274 (63 %)	138 (32 %)	25 (6 %)			
	Follow-up	289 (66 %)	120 (27 %)	28 (6 %)			
4 Moderate activities	Baseline	142 (33 %)	181 (41 %)	114 (26 %)			
	Follow-up	135 (31 %)	185 (42 %)	117 (27 %)			
5 Lifting or carrying groceries	Baseline	128 (29 %)	184 (42 %)	125 (29 %)			
	Follow-up	114 (24 %)	161 (37 %)	172 (39 %)			
6 Climbing several flights of stairs	Baseline	85 (19 %)	149 (34 %)	203 (46 %)			
	Follow-up	104 (24 %)	161 (37 %)	172 (40 %)			
7 Climbing one flight of stairs	Baseline	31 (7 %)	117 (27 %)	289 (66 %)			
	Follow-up	30 (7 %)	128 (29 %)	279 (64 %)			
8 Bending, kneeling, or stooping	Baseline	57 (13 %)	151 (35 %)	229 (52 %)			
	Follow-up	58 (13 %)	150 (34 %)	229 (52 %)			
9 Walking more than a mile	Baseline	115 (26 %)	129 (30 %)	193 (44 %)			
	Follow-up	126 (29 %)	127 (29 %)	184 (42 %)			
10 Walking several blocks	Baseline	54 (12 %)	95 (22 %)	288 (66 %)			
	Follow-up	68 (16 %)	97 (22 %)	272 (62 %)			
11 Walking one block	Baseline	35 (8 %)	75 (17 %)	327 (75 %)			
	Follow-up	41 (9 %)	73 (17 %)	323 (74 %)			
12 Bathing or dressing yourself	Baseline	11 (3 %)	63 (14 %)	363 (83 %)			
	Follow-up	19 (4 %)	47 (11 %)	371 (85 %)			
Role limitations due to physical health (RP)							
13 Did you cut down on the amount of time you spent on work or other activities?	Baseline	306 (70 %)	131 (30 %)				
	Follow-up	290 (66 %)	147 (34 %)				
14 Did you accomplished less than you would like?	Baseline	259 (59 %)	178 (41 %)				
	Follow-up	254 (58 %)	183 (42 %)				
15 Were you limited in the kind of work or other activities?	Baseline	293 (67 %)	144 (33 %)				
	Follow-up	303 (69 %)	134 (31 %)				
16 Did you have difficulty performing the work or other activities?	Baseline	273 (62 %)	164 (38 %)				
	Follow-up	294 (67 %)	143 (33 %)				
Bodily pain (BP)							
21 How much bodily pain have you had?	Baseline	3 (1 %)	20 (5 %)	97 (22 %)	78 (18 %)	88 (20 %)	151 (35 %)
	Follow-up	7 (2 %)	21 (5 %)	93 (21 %)	95 (22 %)	81 (19 %)	140 (32 %)
22 How much did pain interfere with your normal work?	Baseline	17 (4 %)	27 (6 %)	89 (20 %)	120 (28 %)	184 (42 %)	
	Follow-up	13 (3 %)	23 (5 %)	49 (11 %)	125 (29 %)	227 (52 %)	
Social functioning (SF)							
20 To what extent have your physical health or emotional problems interfered with your normal social activities with family, friends, neighbors, or groups?	Baseline	9 (2 %)	25 (6 %)	43 (10 %)	131 (30 %)	229 (52 %)	
	Follow-up	13 (3 %)	23 (5 %)	49 (11 %)	125 (29 %)	227 (52 %)	
32 How much of the time has your physical health or emotional problems interfered with your social activities?	Baseline	24 (5 %)	36 (8 %)	145 (33 %)	68 (16 %)	164 (38 %)	
	Follow-up	34 (8 %)	41 (9 %)	132 (30 %)	74 (17 %)	156 (36 %)	
Role limitations due to emotional problems (RE)							
17 Did you cut down on the amount of time you spent on work or other activities?	Baseline	195 (45 %)	242 (55 %)				
	Follow-up	175 (40 %)	262 (60 %)				
18 Did you accomplished less than you would like?	Baseline	190 (44 %)	247 (57 %)				
	Follow-up	176 (40 %)	261 (60 %)				
19 Did you do work or other activities less carefully than usual?	Baseline	153 (35 %)	284 (65 %)				
	Follow-up	147 (34 %)	290 (66 %)				
Vitality (VT)							
23 Did you feel full of pep?	Baseline	16 (4 %)	32 (7 %)	105 (24 %)	58 (13 %)	145 (33 %)	81 (19 %)
	Follow-up	21 (5 %)	42 (10 %)	104 (24 %)	60 (14 %)	155 (35 %)	55 (13)
27 Did you have a lot of energy?	Baseline	26 (6 %)	73 (17 %)	133 (30 %)	56 (13 %)	94 (22 %)	55 (13 %)
	Follow-up	35 (8 %)	96 (22 %)	134 (31 %)	53 (12 %)	83 (19 %)	36 (8 %)
29 Did you feel worn out?	Baseline	13 (3 %)	19 (4 %)	48 (11 %)	135 (90 %)	90 (2 %)	132 (30 %)
	Follow-up	11 (3 %)	28 (6 %)	56 (13 %)	147 (34 %)	100 (23 %)	95 (22 %)
31 Did you feel tired?	Baseline	29 (7 %)	52 (12 %)	77 (18 %)	166 (38 %)	61 (14 %)	52 (12 %)
	Follow-up	37 (8 %)	53 (12 %)	106 (24 %)	155 (35 %)	56 (13 %)	20 (7 %)
Health comparison (HC)							
2 Compared to 1 year ago, how would you rate your health in general now?	Baseline	32 (7 %)	83 (19 %)	272 (62 %)	43 (10 %)	7 (2 %)	
	Follow-up	34 (8 %)	69 (16 %)	243 (56 %)	78 (18 %)	13 (3 %)

**Table 4:** Hypothesis tests and parameter estimates of Steps 1–3 from Stage 1

	Step 1	Step 2			Step 3									
	BVN	*df*	Chisq_diff_	*p*	Thresholds					Means^a^			SDs^a^	$\rho$
					1	2	3	4	5	Pre	Post	Pre	Post	
MH														
24	✓	3	4.14	0.25	−1.96	−1.41	−0.90	0.19	0.85	3.23	3.83	1.74	1.85	0.59
25	✓	3	0.59	0.90	−2.34	−1.84	−1.14	−0.63	0.10	4.70	4.75	2.19	1.84	0.61
26^b^	✓	3	15.6	<0.01										
26_pre_				−1.62	−0.92	−0.23	0.16	1.20						
26_post_				−1.69	−1.03	−0.23	0.03	1.24						
28	✓	3	5.52	0.14	−2.16	−1.64	−1.16	−0.13	0.60	4.09	4.24	1.96	1.90	0.53
30	✓	3	5.41	0.14	−1.68	−1.28	−0.51	−0.21	0.62	4.40	4.12	2.61	2.47	0.64
GH														
1	✓	2	3.61	0.16	−1.31	−0.07	1.10	1.65		1.08	1.04	0.90	0.71	0.62
33	✓	2	3.63	0.16	−1.62	−1.17	−0.23	0.14		3.72	3.40	2.32	2.05	0.55
34	✓	2	4.88	0.09	−0.77	−0.10	0.52	1.13		1.19	1.11	1.56	1.41	0.49
35	✓	2	2.25	0.32	−1.34	−0.79	0.31	0.72		2.39	2.46	1.91	1.72	0.56
36	✓	2	4.91	0.09	−0.53	−0.07	0.44	1.26		1.22	1.07	2.29	2.02	0.62
PF														
3	✓		n/a		0.37	1.55				−0.26	−0.38	0.80	0.91	0.60
4	✓		n/a		−0.48	0.63				0.42	0.45	0.91	0.89	0.65
5	✓		n/a		−0.59	0.59				0.49	0.51	0.90	0.79	0.72
6	✓		n/a		−0.79	0.18				0.91	0.73	1.05	1.02	0.74
7	✓		n/a		−1.48	−0.39				1.40	1.31	0.95	0.88	0.71
8	✓		n/a		−1.12	−0.06				1.06	1.06	0.94	0.95	0.73
9	✓		n/a		−0.60	0.17				0.81	0.74	1.28	1.32	0.74
10	✓		n/a		−1.08	−0.36				1.55	1.45	1.34	1.43	0.70
11	✓		n/a		−1.36	−0.65				1.91	1.95	1.36	1.48	0.67
12	✓		n/a		−1.78	−0.98				1.96	2.52	1.00	1.47	0.66
RP														
13	n/a		n/a		0.47					−0.53	−0.42	1.00	1.00	0.52
14	n/a		n/a		0.22					−0.23	−0.21	1.00	1.00	0.51
15	n/a		n/a		0.47					−0.44	−0.51	1.00	1.00	0.55
16	n/a		n/a		0.38					−0.32	−0.45	1.00	1.00	0.49
BP														
21	✓	3	9.77	0.02	−2.34	−1.53	−0.55	−0.84	0.41	2.92	2.85	1.18	1.28	0.55
22	✓	2	0.58	0.75	−1.74	−1.23	−0.56	0.11		3.63	2.85	2.06	1.28	0.51
SF														
20	✓	2	1.48	0.48	−1.98	−1.38	−0.90	−0.06		3.28	3.28	1.61	1.71	0.42
32	✓	2	3.09	0.21	−1.51	−1.02	−0.05	0.33		3.16	3.06	1.98	2.15	0.48
RE														
17	n/a		n/a		−0.19					0.14	0.25	1.00	1.00	0.52
18	n/a		n/a		−0.21					0.16	0.25	1.00	1.00	0.60
19	n/a		n/a		−0.40					0.39	0.42	1.00	1.00	0.47
VT														
23	✓	3	6.67	0.08	−1.74	−1.17	−0.31	0.04	0.99	3.18	2.90	1.77	1.72	0.56
27	✓	3	1.05	0.79	−1.48	−0.66	0.18	0.52	1.26	1.93	1.68	1.24	1.21	0.58
29	✓	3	3.46	0.33	−1.89	−1.43	−0.86	0.07	0.64	4.36	3.95	2.31	2.08	0.45
31	✓	3	5.86	0.12	−1.46	−0.83	−0.27	0.77	1.32	2.47	2.16	1.64	1.64	0.52
HC														
2	✓	2	6.96	0.03	−0.68	1.07	1.96			1.77	1.97	1.22	1.35	0.03

*BVN* bivariate normality; the underlying assumption of bivariate normality was evaluated for each item, and considered to be tenable (✓) if the assumption holds for all item pairs according to the RMSEA (see Table 1)

Thresholds were estimated to be equal across measurement occasions using the standard parameterization, where the means and variances of the underlying variables at two consecutive measurement occasions are then defined by: *μ*_1_ + *μ*_2_ = 0 and $\sigma_{1}^{2} + \sigma_{2}^{2} = 2$

*n/a* not applicable, see also Table 1. *MH* mental health, *GH* general physical health, *PF* physical functioning, *RP* role limitations due to physical health, *BP* bodily pain, *SF* social functioning, *RE* role limitations due to emotional health, *VT* vitality, and *HC* health comparison

^a^The alternative parameterization was used to estimate the means and standard deviations of the underlying variables under equal thresholds that were used for subsequent analyses. This entails that identification of the model is achieved by fixing the first two threshold values at zero and one, instead of restricting the sum of the means and variances of the underlying variables. This parameterization is equivalent to the standard parameterization; the linear transformation of the estimates is described in detail by Jöreskog [21]

^b^The means and standard deviations of the underlying variables of Item 26 are not given as they cannot be readily compared across measurements due to recalibration response shift

**Table 5:** Goodness of overall model fit and difference in model fit of the models in Stage 2

Model	*df*	*χ* ^2^	RMSEA [90 % CI]	ECVI [90 % CI]	Compared to	*df*_diff_	CHISQ_diff_	ECVI_diff_ [90 % CI]
Mental health (MH)								
1a Measurement model	25	61.559	0.058 [0.040; 0.076]	0.279 [0.235; 0.341]				
1b No response shift model	31	158.28	0.097 [0.082; 0.112]	0.386 [0.304; 0.485]	Model 1a	6	96.72	0.194 [0.123; 0.276]
1c Response shift model	28	62.979	0.054 [0.036; 0.071]	0.268 [0.224; 0.330]	Model 1a	3	1.320	−0.011 [−0.007; 0.003]
General physical health (GH)								
2a Measurement model	29	61.286	0.047 [0.031; 0.063]	0.162 [0.115; 0.227]				
2b No response shift model	37	72.601	0.051 [0.033; 0.068]	0.173 [0.130; 0.233]	Model 2a	8	11.32	−0.011 [−0.018; 0.019]
Physical functioning (PF)^a^								
3a Measurement Model	151	339.06	0.053 [0.046; 0.061]	1.048 [0.935; 1.180]				
3b No response shift model	169	477.64	0.065 [0.058; 0.072]	1.284 [1.143; 1.442]	Model 3a	18	380.7	0.791 [0.654; 0945]
3c Response shift model	166	374.98	0.054 [0.047; 0.061]	1.062 [0.942; 1.200]	Model 3a	15	46.75	0.038 [−0.001; 0.095]
Role limitations due to physical health (RP)								
4a Measurement model	15	29.727	0.048 [0.021; 0.072]	0.165 [0.138; 0.210]				
4b No response shift model	18	72.543	0.083 [0.064; 0.104]	0.249 [0.120; 0.318]				
4c Response shift model	17	51.313	0.068 [0.047; 0.090]	0.205 [0.164; 0.263]				
Bodily pain (BP)								
5a Measurement model	1	1.798	0.043 [0; 0.143]	0.045 [0.044; 0.064]				
5b No response shift model	3	39.766	0.168 [0.124; 0.216]	0.123 [0.085; 0.179]	Model 5a	2	37.968	0.078 [0.040; 0.133]
5c Response Shift Model	2	5.941	0.067 [0; 0.133]	0.073 [0.038; 0.125]	Model 5a	1	4.143	0.005 [−0.002; 0.029]
Social functioning (SF)								
6a Measurement Model	1	0.143	0 [0; 0.092]	0.042 [0.044; 0.052]				
6b No response shift model	2	1.303	0 [0; 0.084]	0.040 [0.041; 0.055]	Model 6a	1	1.16	−0.002 [−0.002; 0.015]
Role limitations due to emotional problems (RE)								
7a Measurement model	5	13.022	0.061 [0.021; 0.102]	0.103 [0.087; 0.137]				
7b No response shift model	7	17.834	0.060 [0.026; 0.095]	0.105 [0.085; 0.143]				
Vitality (VT)								
8a Measurement model	11	4.7300	0 [0; 0.009]	0.126 [0.140; 0.141]				
8b No response shift model	17	12.326	0 [0; 0.030]	0.116 [0.126; 0.141]	Model 8a	6	7.596	−0.010 [−0.014; 0.016]

*N* = 437; overall model fit and difference in fit was evaluated using WLS Chi-square values that are provided in the standard LISREL output (denoted C2_NNT)

^a^For the subscale PF the WLS Chi-square values did not appear stable, and overall model fit was therefore evaluated using the Satorra–Bentler mean adjusted Chi-square values (denoted C3 in the standard LISREL output), and difference of model fit was evaluated using the difference in uncorrected (DWLS) Chi-square values (denoted C1 in the standard LISREL output)

**Table 6:** Assessment of change in the items of the SF-36: results from Step 4 of Stage 1 and Stage 2, expressed as effect sizes (standardized differences)

Item	Stage 1	Stage 2			
	Observed change in variables *x* ^a^	True change in underlying variables y*	Modeled change in variables y*	Response shift change	True change
Mental health (MH)					
24	0.33**	0.37**	0.36**	0.30^c^**/0.01^d^	0.04
25	0.12*	0.03	0.06		0.06
26^b^	0.06				
28	0.08	0.08	0.05		0.05
30	−0.08	−0.13*	−0.13*	−0.16^c^**	0.03
General physical health (GH)					
1	−0.08	−0.05	−0.08		−0.08
33	−0.08	−0.15*	−0.04		−0.04
34	−0.06	−0.06	−0.07		−0.07
35	0.05	0.04	−0.05		−0.05
36	−0.08	−0.08	−0.11*		−0.11*
Physical functioning (PF)					
3	−0.04	−0.15*	−0.04	−0.00^d^	−0.04
4	0.03	0.04	−0.04		−0.04
5	0.02	0.02	−0.04		−0.04
6	−0.17**	−0.24**	−0.05		−0.05
7	−0.04	−0.12*	−0.05		−0.05
8	0.00	0.00	−0.05		−0.05
9	−0.06	−0.08	−0.05		−0.05
10	−0.10*	−0.10*	−0.06		−0.06
11	−0.04	0.03	−0.05		−0.05
12	0.00	0.51**	0.46**	0.51^c^**/−0.02^d^	−0.03
Role limitations due to physical health (RP)					
13	0.07	0.11*	0.02	0.08^c^	−0.06
14	0.02	0.03	−0.06		−0.06
15	−0.04	−0.07	−0.07		−0.07
16	−0.09	−0.13*	−0.06		−0.06
Bodily pain (BP)					
21	−0.07	−0.06	−0.06	−0.23**	0.17**
22	0.08	0.16**	0.16**		0.16**
Social functioning (SF)					
20	−0.03	0.00	−0.04		−0.04
32	−0.06	−0.05	−0.03		−0.03
Role limitations due to emotional problems (RE)					
17	0.08	0.12*	0.09		0.09
18	0.06	0.09	0.10*		0.10*
19	0.02	0.04	0.08		0.08
Vitality (VT)					
23	−0.13*	−0.17**	−0.19**		−0.19**
27	−0.20**	−0.22**	−0.27**		−0.27**
29	−0.14*	−0.18**	−0.16**		−0.16**
31	−0.18**	−0.20**	−0.20**		−0.20**
Health comparison (HC)					
2	0.11*	0.11*			

*N* = 437; standardized mean differences of 0.2, 0.5, and 0.8 indicate small, medium, and large differences [12]

* *p* < .05; ** *p* < .01

^a^Observed change was calculated by considering the ordinal discrete response scale as a proxy for an interval response scale, and comparing baseline and follow-up measurements using paired *t* tests

^b^Results of Stage 2 for Item 26 cannot be interpreted because recalibration response shift was detected for this item in Stage 1

^c^Recalibration

^d^Reprioritization

#### Mental health (MH): Stage 1

Results of Step indicated that the hypothesis of underlying bivariate normal distribution was tenable for all item pairs. In Step 2, equality constraints on thresholds across measurements lead to a significant deterioration in fit for Item 26 (“Have you felt calm and peaceful?”) (see Table 4). As it is not possible to impose equality restrictions on individual threshold parameters in PRELIS, we could not evaluate whether the non-invariance of thresholds could be attributed to specific thresholds. To evaluate the differences in thresholds of Item 26, we compared the freely estimated threshold at both measurement occasions. Inspection of threshold estimates showed that three out of five thresholds were lower at the second measurement occasion as compared to the first measurement occasion (see Table 4). This indicates recalibration response shift, where it was relatively easy for patients to score high on feeling calm and peaceful after treatment, compared to before treatment. All thresholds for Item 26 were set free to be estimated at both measurement occasions and the item was excluded from further response shift detection analyses in Stage 2. For all other items of MH, means and variances and covariances of the underlying variables were estimated under the restriction of equal thresholds across occasions.

In Step 4, inspection of the estimated mean differences of the underlying variables as compared to the observed mean differences showed that true change in Items 24 and 30 was significant and somewhat larger than the observed change; there was an improvement in the scores of Item 24 and a deterioration in the scores of Item 30 (see Table 6). True change in Item 25 was smaller than the observed change and not significant, and both observed and true change of Items 28 was not significant. There was no significant observed change in Item 26. True change of Item 26 is not given as it cannot be interpreted because the underlying variables have a different scale of measurement.

#### Mental health (MH): Stage 2

The estimated means, variances and covariances of the underlying continuous variables from Step 3 in Stage 1 were used for subsequent analyses in Stage 2. In Step 1, the measurement model yielded reasonable approximate fit (Model 1a, Table 4) and included a residual covariance between Item 26 (“Have you felt calm and peaceful?”) and Item 30 (“Have you been a happy person?”). This indicates that these items have something more in common than is captured by the common factor MH.

In Step 2, invariance restrictions on intercepts and factor loadings were imposed for all items except Item 26. The no response shift model yielded a significant deterioration in model fit as compared to the measurement model, according to both the Chi-square difference test and the ECVI difference test (see Table 5), indicating the presence of response shift.

In Step 3, three response shift effects were detected. Recalibration response shift of Item 24 (“Have you been a nervous person?”) was detected [CHISQ_diff_(1) = 54.8, *p* < .001], where the intercept was higher at follow-up than at baseline. Because items were scored such that higher scores indicate better health, the difference in intercepts indicates that it became relatively difficult to score high on nervousness after antineoplastic treatment, compared to the other items of MH. In addition, reprioritization response shift of the same item was detected [CHISQ_diff_(1) = 28.7, *p* < .001], where the value of the factor loading was higher at follow-up than at baseline. This indicates that the item became more indicative of MH after treatment. Recalibration response shift of Item 30 (“Have you been a happy person?”) was detected [CHISQ_diff_(1) = 11.8, *p* < .001], where the intercept was higher at baseline than at follow-up. This indicates that it became relatively difficult to score high on happiness after treatment, as compared to the other items of MH.

The response shift model, in which all apparent response shifts are taken into account, showed reasonable approximate fit according to the RMSEA, and equivalent model fit as compared to the measurement model (see Table 6). Results of Step 4 indicated that patients showed a significant improvement of MH (change = 0.06, *p* < .001; *d* = 0.08). Before taking into account response shift effects, the change was in the same direction and also significant (change = 0.05, *p* < .001; *d* = 0.08).

Estimates of decomposition of change are presented in Table 6. In general, modeled change in Stage 2 was similar to true change estimates from Stage 1. The estimated true change in Stage 2 showed small improvements in all items, although they were non-significant. Recalibration response shifts in Items 24 and 30 caused the observed improvement (*d* = 0.30) and deterioration (*d* = −0.16), respectively. Results of decomposition of change for Item 26 are not reported because interpretation is hindered due to the difference in measurement scales of the item across occasions.

#### General physical health (GH): Stage 1

The hypothesis of underlying bivariate normal distribution and the equality restrictions on thresholds across measurements were tenable for all pairs of items (see Table 4). In general, true change in the underlying variables was similar to that of observed change, although only the deterioration in true change of Item 33 was significant (see Table 6).

#### General physical health (GH): Stage 2

The measurement model of GH showed reasonable approximate fit (model 2a, Table 5). The no response shift model did not yield a significant deterioration in model fit, indicating that there was no evidence for response shift effects (see Table 5). Overall, patients showed a significant deterioration of GH (change = −0.10, *p* < .001; *d* = −0.19) and also in the items of GH, but only the deterioration in Item 36 was significant (*d* = −0.11; see Table 6).

#### Physical functioning (PF): Stage 1

The hypotheses of underlying bivariate normal distributions were tenable for all item pairs. Equality of thresholds across measurement occasions could not be evaluated, as items with three categories do not provide enough information to test the difference in LR test statistic (see also Table 1). Estimated true change was largely similar to observed change, with significant deterioration in Items 3, 6, 7, and 10. A notable difference occurred for the true change estimate of Item 12, which showed a significant improvement (*d* = 0.51) that was not found for observed change.

#### Physical functioning (PF): Stage 2

The measurement model of PF was modified to include residual covariances between Item 4 (“moderate activities”) and Item 5 (“lifting or carrying groceries”), and between Item 6 (“climbing several flights of stairs”) and Item 7 (“climbing one flight of stairs”). The measurement model that included these residual covariances showed reasonable approximate fit, and the close fit hypothesis could not be rejected (model 3a, Table 5).

The no response shift model fitted worse than the model without across measurement constraints (see Table 5), indicating the presence of response shift. Recalibration response shift of Item 12 (“bathing or dressing yourself”) was detected [CHISQ_diff_(1) = 173.7, *p* < .001], where the intercept was higher at follow-up than at baseline. Thus, patients scored higher on Item 12 after treatment, relative to the other items of PF. Because higher scores on Item 12 are indicative of fewer limitations, it became relatively difficult to endorse limitations on this item after antineoplastic treatment. In addition, reprioritization response shift of Item 12 (“bathing or dressing yourself”) and Item 4 (“moderate activities”) was detected [CHISQ_diff_(1) = 146.2, *p* < .001; CHISQ_diff_(1) = 14.0, *p* < .001], where the factor loadings of both items were higher at follow-up as compared to baseline, indicating that both items became more indicative of PF after treatment.

The response shift model yielded reasonable approximate fit according to the RMSEA, and equivalent approximate model fit as compared to the measurement model (see Table 5). Patients showed no significant change in PF (change = −0.05, *p* = .13), but before taking into account response shift effects the change was in the opposite direction and significant (change = 0.02, *p* = .041). Therefore, not taking into account response shift effects would have overestimated changes in PF.

Inspection of change estimates for individual items showed (non-significant) deterioration in all items. However, for Item 12 there was a significant improvement due to recalibration response shift (*d* = 0.51).

#### Role limitations due to physical health (RP): Stage 1

As RP consists of dichotomous items, the hypothesis of bivariate normality and equality of thresholds across measurement occasions could not be evaluated (see Table 1). Inspection of true change estimates revealed a significant improvement of Item 13 and a significant deterioration of Item 16 (see Table 6).

#### Role limitations due to physical health (RP): Stage 2

The measurement model of RP showed close approximate fit (model 4a, Table 5). To enable the investigation of response shift with dichotomous items, the no response shift model requires some adaptations (i.e., additional scaling parameters; see Supplement 1.4 for more details). As a result, only recalibration response shift can be investigated with dichotomous items, and the presence of recalibration response shift is evaluated based on overall goodness-of-fit of the no response shift model. The overall model fit of the no response shift model of RP was not good (model 4b, Table 5), indicating the presence of response shift. Recalibration response shift of Item 13 (“Did you cut down on amount of time you spent on work or other activities?”) was detected [CHISQ_diff_(1) = 21.2, *p* < .001], where the intercept was higher at follow-up than at baseline. Patients scored higher on Item 13 after treatment, relative to the other items of RP. Because higher scores on Item 13 are indicative of fewer limitations, it became relatively difficult to endorse limitations on this item after antineoplastic treatment. The response shift model that included this recalibration response shift showed an improvement in overall model fit as compared to the no response shift model, and reasonable approximate fit according to the RMSEA (see Table 5).

Inspection of common factor means showed no significant change of RP (change = −0.07, *p* = .15; *d* = −0.07). Taking into account recalibration response shift did not affect the interpretation of change. Inspection of change estimates for individual items showed (non-significant) deterioration for all items and that the improvement in Item 13 was explained by recalibration (see Table 6).

#### Bodily pain (BP): Stage 1

The hypotheses of underlying bivariate normal distributions were tenable for all pairs of items. The equality restrictions on thresholds across measurements showed a significant deterioration in fit for Item 21 according to the Chi-square difference test (*p* = .02, see Table 4), but the ECVI difference test showed no significant deterioration in approximate fit (ECVI_diff_ = 0.009, 90 % CI −0.005 to 0.040). Inspection of true change estimates showed a (non-significant) deterioration in Item 21, whereas Item 22 showed a significant improvement (see Table 6).

#### Bodily pain (BP): Stage 2

To achieve identification of the measurement model of the two-item BP subscale, we applied the constraint of zero residual covariances as this restriction yielded best model fit (see Supplement 1.3 for more details). The measurement model showed exact fit, but comparison with the no response shift model showed evidence of response shift (see Table 5). Investigation of response shift effects showed that the model could be improved by freeing the restrictions on the intercepts, indicating recalibration response shift. We chose to free the intercept of Item 21 “level of pain,” where it became relatively difficult to score high on this item after treatment as compared to the item “interference of pain.” The response shift model showed equivalent approximate fit as compared to the measurement model. Inspection of common factor means showed a small but non-significant improvement of BP (change = 0.18, *p* = .09; *d* = 0.19). Before taking into account response shift, the improvement in BP was slightly smaller, but significant (change = 0.13, *p* < .001; *d* = 0.14).

Inspection of change estimates for the two individual items showed that the difference in behavior of both items was explained by recalibration of Item 21 (*d* = −0.23), whereas the modeled change showed significant improvement for Item 22 (*d* = 0.16) but no significant change for Item 21 (see Table 6).

#### Social functioning (SF): Stage 1

The hypotheses of underlying bivariate normal distributions and the equality restrictions on thresholds across measurements were tenable for both items. Estimates of true change showed no significant differences (see Table 6).

#### Social functioning (SF): Stage 2

To achieve identification of the two-item measurement model of SF we applied the constraint of equal factor loadings for both items at each measurement occasion, as this restriction yielded best model fit (see Supplement 1.3 for more details). Both the measurement model and the no response shift model of SF showed exact fit (models 6a and 6b, Table 5), and there was no evidence for response shift. Inspection of common factor means showed a small but significant deterioration of SF (change = −0.05, *p* < .001; *d* = −0.05), although the change estimates for individual items were not significant (see Table 6).

#### Role limitations due to emotional health (RE): Stage 1

Because the subscale RE consists of dichotomous items, the hypothesis of bivariate normality and equality of thresholds across measurement occasions could not be evaluated. Both observed and true change showed improvements for all items, although only the estimated true change for Item 17 was significant (see Table 6).

#### Role limitations due to emotional health (RE): Stage 2

Both the measurement model and the no response shift model of RE yielded reasonable approximate fit (model 5a and model 5b, Table 5). Therefore, there was no evidence of (recalibration) response shift (see Supplement 1.4). Inspection of common factor means showed no significant change of RE (change = 0.09, *p* = .09; *d* = 0.10), but Item 17 showed a significant improvement (see Table 6).

#### Vitality (VT): Stage 1

The hypotheses of underlying bivariate normal distributions and the equality restrictions on thresholds across measurements were tenable for all item pairs. The estimated true change was similar to that of observed change, although true change estimates were slightly larger. All items showed a significant deterioration (see Table 6).

#### Vitality (VT): Stage 2

The measurement model included a residual covariance between Item 29 (“Did you feel worn out?”) and Item 31 (“Did you feel tired?”), and showed exact fit (model 6a, Table 5). The no response shift model also yielded exact fit, and equivalent model fit as compared to the measurement model, indicating no evidence of response shift (see Table 5). Overall, patients showed a significant deterioration of VT (change = −0.27, *p* < .001; *d* = −0.34) and also a significant deterioration in all individual items (see Table 6).

#### Health comparison (HC): Stage 1

The subscale HC consists of only one item, so we can only conduct Stage 1 analyses. Evaluation of bivariate normality showed that this hypothesis was tenable, and although the restriction of equality of thresholds across measurement occasions showed a significant deterioration according to the Chi-square difference test (*p* = 0.03, see Table 3), there was no significant deterioration in approximate model fit (ECVI_diff_ = 0.007, 90 % CI −0.004 to 0.035). There was a significant improvement across measurement occasions for both observed and true change (see Table 4).

## Discussion

In this paper we explained how the SEM approach for detection of response shift and assessment of true change can be applied to discrete data by assuming underlying continuous variables with bivariate normal distributions (Stage 1), and how the resulting estimates can be used in a common factor model (Stage 2). The proposed SEM approach thus enables the detection of response shift and assessment of true change in discrete ordinal responses.

### Substantive interpretation of results

We applied the proposed SEM approach to all items of the SF-36. In our sample of cancer patients, we found that the model of underlying bivariate normal distributed continuous variables was tenable for all items (Stage 1). We detected recalibration response shift in the item “Have you felt calm and peaceful?” of the MH subscale, where it was relatively easy for patients to score high on feeling calm and peaceful after treatment, as compared to before treatment. We assessed change in the underlying variables and found that estimated true change was mostly similar to observed change, although estimated true change was somewhat larger in general. When change of the observed variables would be assessed as if they have interval scales (i.e., without taking into account their discrete properties), there would be ten items that showed significant change. Whereas true change estimates showed significant change in 18 items. Moreover, only for one item the results of true change no longer showed a significant difference between measurement occasions. Taken together, these results indicate that the model of Stage 1 can be used to provide an informative assessment of change. Furthermore, the estimates of the model can be used to enable detection of response shift and assessment of true change in Stage 2.

In Stage 2, we used a common factor model to detect response shift and assess true change in each subscale of the SF-36 separately. Results showed that patients’ MH improved, while their physical health, VT and SF deteriorated. No change was found for PF, role limitations due to physical health, role limitations due to emotional problems and BP. In general, when asked to compare their current health state to their health state the previous year, patients indicated that their health had improved.

Response shift effects were detected in individual items of the subscales MH, PF, role limitations due to physical health and BP. For the MH subscale, recalibration and reprioritization response shift was detected in the item “nervousness”, where it became relatively difficult to score high on nervousness after antineoplastic treatment as compared to the other items, and nervousness became more important to the measurement of MH. An explanation for this result could be that the start of treatment causes patients to experience less nervousness relative to the other indicators of MH. In addition, it might be that the decreased nervousness becomes especially relevant for patients’ mental state. Recalibration response shift was also detected in the item about “happiness”, where it became relatively difficult to score high on happiness after antineoplastic treatment. Thus, it seems that even though patients’ MH improved over time, this improvement was not found to the same degree for patients’ happiness as compared to the other indicators of MH. Not taking into account response shift effects would have led to an underestimation of change in MH.

For the PF subscale recalibration and reprioritization response shift was detected for the item “bathing or dressing oneself”, where it became relatively difficult to endorse limitations with bathing and/or dressing oneself after antineoplastic treatment as compared to the other items, and the item became more important for the measurement of PF. In addition, the item “moderate activities” also became more important for the measurement of PF after treatment. Therefore, it seems that being able to execute these moderate and personal activities becomes more important for patients’ PF after treatment as compared to the other items. In addition, even though patients’ PF did not change, limitations with regard to bathing or dressing oneself showed an improvement across time. Not taking into account response shift effects would have led to an overestimation of change of PF.

For the subscale role limitations due to physical health recalibration response shift of the item “time for work and other activities” was detected, where it became relatively difficult to endorse limitations on this item after antineoplastic treatment. Thus, even though patients’ overall role limitations due to physical health did not change, it seems that patients experienced decreased limitations with regard to time for work and other activities. A possible explanation for this result could be that patients get used to changes with regard to the allocation of available time, or adapt to the possible limitations due to their physical condition.

Finally, for BP recalibration response shift was detected. As this scale consist of only two items, detection of response shift indicates that the two items of this subscale behave differently. In our example, patients indicated to experience relatively fewer limitations due to their experienced pain as compared to the level of experienced pain. A possible explanation for this result could be that patients get used to or adapt to the experienced limitations due to their physical condition.

Compared to the selected study sample, the group of patients that was excluded due to attrition or due to too many missing values showed lower Karnofsky performance and more progressive tumors. Therefore, it should be noted that the results of our study may not be generalizable to the full population.

Taken together, these results provide information about the behavior of individual items within each subscale of the SF-36. Specifically, the results give insight as to what extent changes at the item level can be attributed to changes at subscale level (e.g., MH or PF), and which items show response shift. To our knowledge, this is the first time that response shift has been investigated in all individual items of the SF-36 questionnaire—one of the most widely used measurement instruments in the literature of HRQL. Although item-level data have been considered in previous research of the SF-36 [2, 17], response shift was only investigated in the items of a single subscale [17], or response shift in all items was tested globally instead of in individual items [2]. Therefore, the application of the SEM approach for discrete data to the items of the SF-36 in the present paper provides a substantive contribution to the literature on response shift phenomena.

### Limitations of the proposed SEM approach

In the application of the SEM approach for discrete data, the question arises when to treat item responses as discrete ordinal responses and when to treat them as continuous responses. Item response scales are usually discrete as they only have limited number of response categories. However, when the number of response categories is larger (e.g., seven or more), discrete ordinal responses can be considered to sufficiently approximate continuous interval scales, so that statistical analyses for interval variables may be appropriate [15]. The treatment of discrete item responses should therefore be based on both substantive considerations (e.g., can the underlying measurement scale be considered continuous?) and statistical considerations (e.g., does the distribution of scores of the observed variables approximate a normal distribution? Are the chosen statistical techniques appropriate?). In the present paper, we applied the SEM approach for discrete data to ordinal item responses with different numbers of response categories (i.e., two, three, five and six). In our example, we considered the measurement scale of all items to be discrete. By definition, univariate normality does not hold for discrete variables. However, the proposed SEM approach has the flexibility to include not only variables with different numbers of response categories, but also variables with different measurement scales (e.g., the PRELIS program can be used to calculate the appropriate correlations between the variables) and could even be applied to non-ordinal binary data.

In Stage 1, we test the assumption of underlying bivariate normality and derive estimates of polychoric correlations, variances and means of the underlying variables under equal thresholds across measurement occasions. Stage 1 also provides information on the detection of response shift, in addition to the usual detection of response shifts in Stage 2. Recalibration response shift in Stage 1 can be interpreted as scale recalibration relative to the scale of the underlying continuum, whereas recalibration response shift in Stage 2 can be interpreted as scale recalibration relative to the scale of the common factor (and thus relative to the other variables measuring the same common factor).

It should be noted that under some circumstances it is not be possible to detect recalibration response shift. First, invariance of thresholds can only be evaluated when the number of response categories is larger than three, for variables with fewer response categories invariance of thresholds is assumed to hold. Second, non-invariance of thresholds might not be detected if the thresholds differ by an additive constant (this would be captured by mean differences in the underlying variables) or a multiplicative constant (this would be captured by differences in the standard deviations of the underlying variables). Similarly, non-invariance of intercepts in Stage 2 might not be detected if the intercepts differ by an additive constant (this would be captured by mean differences in the common factors) or a multiplicative constant (this would be captured by differences in the standard deviations of the common factors).

Although it might be possible to investigate whether differences in thresholds can be attributed to specific threshold parameters, this was not applied in the present paper because it is not possible to impose equality restrictions on individual thresholds in the PRELIS program that was used for statistical analyses. It might be of substantive interest to further investigate non-invariance of specific thresholds, but it does not resolve the fact that the scales of the underlying variables are different. It might also be of substantive interested to test more restrictive hypotheses about thresholds, such as the hypothesis of equally spaced thresholds (e.g., the difference between different answer categories in terms of the underlying variables are equal).

Although the performance of the common factor model and the estimation of polychoric correlations are reasonably robust against moderate violations of normality (e.g., [3, 9, 13]), similar studies on the performance of the common factor model under violations of invariant thresholds are needed. Millsap and Yun-Tein [23] investigated the impact of non-invariant thresholds in a multigroup context and concluded that when invariant thresholds are erroneously imposed, group differences in thresholds may be mistaken for group differences in residual variances. It would be interesting to perform a simulation study with the proposed methods for response shift detection and investigate the impact of (violations of) threshold invariance, number of response categories, number of variables in the common factor model, size of the bias, sample size, missing data, etc. Such a simulation study would be helpful to further substantiate the appropriateness of the proposed SEM approach for discrete data under different circumstances.

The SEM approach for discrete data was applied to the individual items of each subscale of the SF-36 separately. A limitation of this approach is that it does not allow for detection of reconceptualization response shift due to other factors, such as other subscales or demographic or clinical variables. However, the proposed approach can be extended to enable the detection of reconceptualization response shift due to these factors. For example, it would be interesting to investigate response shift in all the items of the SF-36 simultaneously by using one common factor model that includes all eight multi-item subscales, and the one-item scale of health comparison. However, it should be noted that such highly complex models require much larger samples in order for the proposed methods to work appropriately. As an alternative strategy one might conduct pairwise analyses of subscales, to reduce the model complexity while still enabling the investigation of reconceptualization response shift due to another subscale. A similar approach could also be used to investigate the effects of possible explanatory or confounding variables (e.g., gender, age, type of disease, or treatment modality). In the present paper, we chose to investigate all subscales separately to enable the explanation of the proposed methods for various situations (i.e., different number of response categories and different number of items per scale) and facilitate the analyses and interpretation of results (i.e., more parsimonious models). Further extensions of the proposed methods that include more measurement occasions, other subscales, or explanatory variables, would be an interesting topic for future research.

SEM with discrete data can be done using standard statistical computer programs [18, 28, 31, 34]. However, differences exist between programs in how they handle the analyses of discrete data. For example, the underlying assumptions of Stage 1 (i.e., bivariate normality and equal thresholds) are usually not tested but assumed to hold. Moreover, not all computer programs make an explicit distinction between the estimation of polychoric correlations and the fitting of structural equation models to the polychoric correlations. Some programs might test invariance of thresholds as an alternative to the invariance of intercepts (e.g., see [23]), and as a consequence test conceptually different hypotheses (i.e., differences in thresholds are conceptually different from differences in intercepts). In addition, different programs may use different (default) corrections for the resulting Chi-square values, and different options for evaluation of overall goodness-of-fit and differences in model fit may lead to different results. For the present paper, analyses were performed using the PRELIS (Stage 1) and LISREL (Stage 2) programs [20]. With PRELIS it is possible to evaluate the Stage 1 model for discrete data. In Stage 2, the WLS Chi-square value was used to evaluate model fit, as it provides a valid test statistic under non-normality and has the convenient property that it can also be used for the calculation of approximate fit indices and for the comparison of nested models. However, when sample sizes are small or models are large, the performance of the WLS test statistic might not be stable and one might consider alternative adjustment to the Chi-square statistics (see also Supplement 1.2). One should be aware that there are notable differences between different computer programs in handling discrete data and that the choice of computer program may also influence the ease with which one can apply the required analyses.

Besides SEM techniques, there are other statistical techniques for the detection of response shift available, such as ordinal logistic regression, the contingency tables methods, and probit regression. Methods relying on item response theory (IRT) analysis are probably the most popular method for the analysis of discrete ordinal data. Factor analysis methods are not conceptually different from IRT methods, but the former are usually applied to continuous data. The relationship between IRT and factor analysis has been described by [26]. Takane and De Leeuw [39] showed that WLS estimation with polychoric correlations in factor analysis is equivalent to fitting the normal ogive model with marginal ML estimation in IRT. However, advantages of SEM are that the models can be easily extended to multidimensional models (e.g., longitudinal models, or models that include multiple subscales) and that the hypothesized dimensional structure of the model can be tested.

## Conclusion

Investigation of response shift and assessment of change at the individual item level can give insight into which items of a subscale contribute to changes at the subscale level, or which items behave differently from the other items. Analyses of items therefore provide different information than analyses of subscales. For example, it could be that there is no change (or no occurrence of response shift) at the subscale level, while there are changes at the level of individual items (or possible response shift effects) that cancel each other out. In addition, item-level analyses enable the identification of items that are most important to changes at the level of the subscale. Although the proposed SEM approach for discrete data needs further scrutiny using simulation studies, it leads to a better understanding of the response shift phenomena and enhances interpretation of change in the area of HRQL.

## Electronic supplementary material

Below is the link to the electronic supplementary material.

Supplementary material 1 (DOCX 19 kb)

Supplementary material 2 (DOCX 21 kb)

Supplementary material 3 (DOCX 32 kb)

Supplementary material 4 (DOCX 22 kb)
