# Supplementary material for: Using structural equation modeling to detect response shifts and true change in discrete variables: an application to the items of the SF-36
Source: Qual Life Res. 2015 Dec 22;25:1361–83. doi: 10.1007/s11136-015-1195-0 (PMC4870306; doi:10.1007/s11136-015-1195-0)
Supplement: Supplementary file 1 — Supplementary material 1 (DOCX 19 kb) [file 11136_2015_1195_MOESM1_ESM.docx]

SUPPLEMENT 1

ADDITIONAL DETAILS OF THE SEM APPROACH FOR THE DETECTION OF RESPONSE SHIFT AND TRUE CHANGE IN DISCRETE DATA

**1.1 Invariance of thresholds across measurement occasions**

**1.2 Alternative estimation methods for discrete data**

**1.3 SEM with two observed variables**

**1.4 SEM with dichotomous variables**

**1.1 Invariance of thresholds across measurement occasions**

When the same variables are measured repeatedly (i.e., in longitudinal assessment) the imposition of invariant thresholds across measurement occasions is required to enable estimation of differences in means and variances of the underlying variables (i.e., **μ***_y*_* and diag(**Σ***_y*_*)). This restriction establishes a common scale, which is a necessary requirement for assessment of change across measurement occasions.

For variables with only one threshold there is not enough information to estimate the variances of the underlying variables, and thus equality restrictions on thresholds across measurement occasions for dichotomous variables only enables estimation of differences in means of the underlying variables.

**1.2 Alternative estimation methods for discrete data**

Application of the ML estimation method to polychoric correlations yield biased standard errors and chi-square test statistics. One of the estimation methods that can be applied in the case of discrete data is the ‘weighted least squares’ (WLS; [5]) method, which uses the ‘asymptotic variance covariance matrix’ to adjust for imprecision of the estimated **Σ***_y*_* and **μ** *_y*_*. This matrix contains the variances and covariances of the estimated polychoric correlations, which can be interpreted as indices of precision of estimation. The inverse of this matrix is used as a weight-matrix in the estimation procedure. However, the estimated weight matrix is unstable when models are complex (e.g., the size of the weight matrix increase rapidly with increasing number of variables) and when the sample size is small (e.g., less than 1000 or even 1600) (see [26], [27], [4]). Therefore, it is often convenient to use the diagonally weighted least squares (DWLS) estimator with robust standard errors, where only the diagonal of the weight matrix is used to yield unbiased parameter estimates, whereas standard errors of parameter estimates are adjusted using the full weight matrix. Simulation studies have shown that the DWLS estimator with robust standard errors performs well in terms of unbiased parameter estimates and precise standard errors (e.g., [12], [13], [38]).

The WLS and robust DWLS estimation methods yield correct parameter estimates, but only the WLS test-statistic yields correct standard errors and an asymptotic chi-square distribution (if the model holds). The WLS chi-square value can therefore also be used for the calculation of approximate fit indices (e.g., RMSEA). Moreover, the WLS chi-square values can be used to compare nested models using a chi-square difference test. When sample sizes are small or models are complex, the performance of WLS might not be stable. In such cases, an alternative adjustment to the (DWLS) chi-square statistic can be used, which requires the calculation of a scaling-factor to correct for non-normality ([32]). Because the resulting statistic is not chi-square distributed, this complicates the evaluation of approximate fit and differences in fit. As available adjustments for difference tests (e.g., [33]) can be computationally demanding and sometimes lead to inadmissible solutions ([8]), it has been proposed to evaluate approximate fit and differences in fit using the uncorrected chi-square values ( [14]).

**1.3 SEM with two observed variables**

When there are only two observed variables (e.g., a scale that consists of only two items) the Measurement Model cannot be tested, as these small numbers of observed variables do not provide enough information to uniquely estimate all model parameters (i.e., the model is not identified). In such cases, additional assumptions are required to enable estimation of the Measurement Model. For example, equality restrictions on factor loadings (i.e., assuming both variables are equally indicative of the underlying common factor) or restricting the across occasion covariances of residual factors to zero (i.e., assuming the systematic relations between observed variables are fully explained by the underlying common factor) can be used to enable estimation of the Measurement Model. The choice between these two identification restrictions can be based on substantive arguments (e.g., that both items are equally indicative of the underlying common factor) or on results of model fit evaluation (i.e., choosing the restriction that yields the best model fit).

In addition, investigation of response shift is complicated by the fact that freeing a restriction on a measurement parameter associated with one variable is equivalent to freeing the same restriction associated with the other variable. Therefore, possible violations of invariance restrictions cannot be attributed to a specific variable on the basis of statistical information, so that the attribution of response shift can only be based on substantive consideration.

**1.4 SEM with dichotomous variables**

For dichotomous variables (i.e., when analyzing a matrix of tetrachoric correlations), the model of No Response Shift requires some adaptations to yield appropriate parameter estimates and hypothesis tests. Specifically, as variances cannot be estimated in step 1, it is necessary to estimate the scaling parameters at the second measurement occasion, to account for differences in variances across measurement occasions. As a result, it is not possible to investigate differences in factor loadings or residual variances. Furthermore, the No Response Shift Model and the Measurement Model cannot be compared using the chi-square difference test, as they are not hierarchically related. As an alternative, the presence of response shift can be evaluated based on overall goodness-of-fit of the No Response Shift Model. When the model does not fit, this indicates the presence of (uniform recalibration) response shift. Due to the necessary estimation of the scaling parameters at the second measurement, only the non-invariance of intercepts (i.e., uniform recalibration response shift) can be investigated.
