# Supplementary material for: Using structural equation modeling to detect response shifts and true change in discrete variables: an application to the items of the SF-36
Source: Qual Life Res. 2015 Dec 22;25:1361–83. doi: 10.1007/s11136-015-1195-0 (PMC4870306; doi:10.1007/s11136-015-1195-0)
Supplement: Supplementary file 2 — Supplementary material 2 (DOCX 21 kb) [file 11136_2015_1195_MOESM2_ESM.docx]

APPENDIX A

STAGE 1: PRELIS SYNTAXES FOR ANALYSES OF ALL ITEMS OF THE SF-36

###########################################################################

# Mental Health

###########################################################################

STEP 1: BIVARIATE NORMALITY

Data Ninputvariables = 10

Labels

m1mh1 m1mh2 m1mh3 m1mh4 m1mh5

m2mh1 m2mh2 m2mh3 m2mh4 m2mh5

Rawdata=MH.RAW RE

Output MA=PM

STEP 2: INVARIANT THRESHOLDS

Data Ninputvariables = 10

Labels

m1mh1 m1mh2 m1mh3 m1mh4 m1mh5

m2mh1 m2mh2 m2mh3 m2mh4 m2mh5

Rawdata=MH.RAW RE

ET m1mh1 m2mh1

ET m1mh2 m2mh2

ET m1mh3 m2mh3

ET m1mh4 m2mh4

ET m1mh5 m2mh5

Output MA=PM

STEP 4: COMPUTE POLYCHORIC CORRELATIONS, VARIANCES AND MEAN VECTOR

Data Ninputvariables = 10

Labels

m1mh1 m1mh2 m1mh3 m1mh4 m1mh5

m2mh1 m2mh2 m2mh3 m2mh4 m2mh5

Rawdata=MH.RAW RE

ET m1mh1 m2mh1

ET m1mh2 m2mh2

!ET m1mh3 m2mh3 !assumption of invariant thresholds did not hold

ET m1mh4 m2mh4

ET m1mh5 m2mh5

Output MA=CM PA PV CM=MH.CM AC=MH.ACC ME=MH.ME TH=MH.TH

###########################################################################

# General Physical Health

###########################################################################

STEP 1: BIVARIATE NORMALITY

Data Ninputvariables = 10

Labels

m1gh1 m1gh2 m1gh3 m1gh4 m1gh5

m2gh1 m2gh2 m2gh3 m2gh4 m2gh5

Rawdata=GH.RAW RE

Output MA=PM

STEP 2: INVARIANT THRESHOLDS

Data Ninputvariables = 10

Labels

m1gh1 m1gh2 m1gh3 m1gh4 m1gh5

m2gh1 m2gh2 m2gh3 m2gh4 m2gh5

Rawdata=GH.RAW RE

ET m1gh1 m2gh1

ET m1gh2 m2gh2

ET m1gh3 m2gh3

ET m1gh4 m2gh4

ET m1gh5 m2gh5

Output MA=PM

STEP 4: COMPUTE POLYCHORIC CORRELATIONS, VARIANCES AND MEAN VECTOR

Data Ninputvariables = 10

Labels

m1gh1 m1gh2 m1gh3 m1gh4 m1gh5

m2gh1 m2gh2 m2gh3 m2gh4 m2gh5

Rawdata=GH.RAW RE

ET m1gh1 m2gh1

ET m1gh2 m2gh2

ET m1gh3 m2gh3

ET m1gh4 m2gh4

ET m1gh5 m2gh5

Output MA=CM CM=GH.CM AC=GH.ACC ME=GH.ME

###########################################################################

# Physical Functioning

###########################################################################

STEP 1: BIVARIATE NORMALITY

Data Ninputvariables = 20

Labels

m1pf01 m1pf02 m1pf03 m1pf04 m1pf05 m1pf06 m1pf07 m1pf08 m1pf09 m1pf10

m2pf01 m2pf02 m2pf03 m2pf04 m2pf05 m2pf06 m2pf07 m2pf08 m2pf09 m2pf10

Rawdata=PF.RAW RE

Output MA=PM

STEP 2: INVARIANT THRESHOLDS -> NOT TESTABLE

Data Ninputvariables = 20

Labels

m1pf01 m1pf02 m1pf03 m1pf04 m1pf05 m1pf06 m1pf07 m1pf08 m1pf09 m1pf10

m2pf01 m2pf02 m2pf03 m2pf04 m2pf05 m2pf06 m2pf07 m2pf08 m2pf09 m2pf10

Rawdata=PF.RAW RE

ET m1pf01 m2pf01

ET m1pf02 m2pf02

ET m1pf03 m2pf03

ET m1pf04 m2pf04

ET m1pf05 m2pf05

ET m1pf06 m2pf06

ET m1pf07 m2pf07

ET m1pf08 m2pf08

ET m1pf09 m2pf09

ET m1pf10 m2pf10

Output MA=PM

STEP 4: COMPUTE POLYCHORIC CORRELATIONS, VARIANCES AND MEAN VECTOR

Data Ninputvariables = 20

Labels

m1pf01 m1pf02 m1pf03 m1pf04 m1pf05 m1pf06 m1pf07 m1pf08 m1pf09 m1pf10

m2pf01 m2pf02 m2pf03 m2pf04 m2pf05 m2pf06 m2pf07 m2pf08 m2pf09 m2pf10

Rawdata=PF.RAW RE

Output MA=CM CM=PF.CM AC=PF.ACC ME=PF.ME

###########################################################################

# Role Limitations due to Physical Health

###########################################################################

STEP 1: BIVARIATE NORMALITY

Data Ninputvariables = 8

Labels

m1rp1 m1rp2 m1rp3 m1rp4

m2rp1 m2rp2 m2rp3 m2rp4

Rawdata=RP.RAW RE

Output MA=PM

STEP 2: INVARIANT THRESHOLDS -> NOT TESTABLE

Data Ninputvariables = 8

Labels

m1rp1 m1rp2 m1rp3 m1rp4

m2rp1 m2rp2 m2rp3 m2rp4

Rawdata=RP.RAW RE

ET m1rp1 m2rp1

ET m1rp2 m2rp2

ET m1rp3 m2rp3

ET m1rp4 m2rp4

Output MA=PM

STEP 4: COMPUTE TETRACHORIC CORRELATIONS AND MEAN VECTOR

Data Ninputvariables = 8

Labels

m1rp1 m1rp2 m1rp3 m1rp4

m2rp1 m2rp2 m2rp3 m2rp4

Rawdata=RP.RAW RE

Output MA=CM KM=RP.KM AC=RP.ACC ME=RP.ME

###########################################################################

# Bodily Pain

###########################################################################

STEP 1: BIVARIATE NORMALITY

Data Ninputvariables = 4

Labels

m1bp1 m1bp2

m2bp1 m2bp2

Rawdata=BP.RAW RE

Output MA=PM

STEP 2: INVARIANT THRESHOLDS

Data Ninputvariables = 4

Labels

m1bp1 m1bp2

m2bp1 m2bp2

Rawdata=BP.RAW RE

ET m1bp1 m2bp1

ET m1bp2 m2bp2

Output MA=PM

STEP 4: COMPUTE POLYCHORIC CORRELATIONS, VARIANCES AND MEAN VECTOR

Data Ninputvariables = 4

Labels

m1bp1 m1bp2

m2bp1 m2bp2

Rawdata=BP.RAW RE

ET m1bp1 m2bp1

ET m1bp2 m2bp2

Output MA=CM CM=BP.CM AC=BP.ACC ME=BP.ME

###########################################################################

# Social Functioning

###########################################################################

STEP 1: BIVARIATE NORMALITY

Data Ninputvariables = 4

Labels

m1sf1 m1sf2

m2sf1 m2sf2

Rawdata=SF.RAW RE

Output MA=PM

STEP 2: INVARIANT THRESHOLDS

Data Ninputvariables = 4

Labels

m1sf1 m1sf2

m2sf1 m2sf2

Rawdata=SF.RAW RE

ET m1sf1 m2sf1

ET m1sf2 m2sf2

Output MA=PM

STEP 4: COMPUTE POLYCHORIC CORRELATIONS, VARIANCES AND MEAN VECTOR

Data Ninputvariables = 4

Labels

m1sf1 m1sf2

m2sf1 m2sf2

Rawdata=SF.RAW RE

ET m1sf1 m2sf1

ET m1sf2 m2sf2

Output MA=CM CM=SF.CM AC=SF.ACC ME=SF.ME

###########################################################################

# Role Limitations due to Emotional Problems

###########################################################################

STEP 1: BIVARIATE NORMALITY

Data Ninputvariables = 6

Labels

m1re1 m1re2 m1re3

m2re1 m2re2 m2re3

Rawdata=RE.RAW RE

Output MA=PM

STEP 2: INVARIANT THRESHOLDS -> NOT TESTABLE

Data Ninputvariables = 6

Labels

m1re1 m1re2 m1re3

m2re1 m2re2 m2re3

Rawdata=RE.RAW RE

ET m1re1 m2re1

ET m1re2 m2re2

ET m1re3 m2re3

Output MA=PM

STEP 4: COMPUTE POLYCHORIC CORRELATIONS, VARIANCES AND MEAN VECTOR

Data Ninputvariables = 6

Labels

m1re1 m1re2 m1re3

m2re1 m2re2 m2re3

Rawdata=RE.RAW RE

Output MA=CM CM=RE.CM AC=RE.ACC ME=RE.ME

###########################################################################

# Vitality

###########################################################################

STEP 1: BIVARIATE NORMALITY

Data Ninputvariables = 8

Labels

m1vt1 m1vt2 m1vt3 m1vt4

m2vt1 m2vt2 m2vt3 m2vt4

Rawdata=VT.RAW RE

Output MA=PM PA XU

STEP 2: INVARIANT THRESHOLDS

Data Ninputvariables = 8

Labels

m1vt1 m1vt2 m1vt3 m1vt4

m2vt1 m2vt2 m2vt3 m2vt4

Rawdata=VT.RAW RE

ET m1vt1 m2vt1

ET m1vt2 m2vt2

ET m1vt3 m2vt3

ET m1vt4 m2vt4

Output MA=PM PA XU

STEP 4: COMPUTE POLYCHORIC CORRELATIONS, VARIANCES AND MEAN VECTOR

Data Ninputvariables = 8

Labels

m1vt1 m1vt2 m1vt3 m1vt4

m2vt1 m2vt2 m2vt3 m2vt4

Rawdata=VT.RAW RE

ET m1vt1 m2vt1

ET m1vt2 m2vt2

ET m1vt3 m2vt3

ET m1vt4 m2vt4

Output MA=CM CM=VT.CM AC=VT.ACC ME=VT.ME

###########################################################################

# Health Comparison

###########################################################################

STEP 1: BIVARIATE NORMALITY

Data Ninputvariables = 2

Labels

m1ht

m2ht

Rawdata=HT.RAW RE

Output MA=PM

STEP 2: INVARIANT THRESHOLDS

Data Ninputvariables = 2

Labels

m1ht

m2ht

Rawdata=HT.RAW RE

ET m1ht m2ht

Output MA=PM

STEP 4: COMPUTE POLYCHORIC CORRELATIONS, VARIANCES AND MEAN VECTOR

Data Ninputvariables = 2

Labels

m1ht

m2ht

Rawdata=HT.RAW RE

ET m1ht m2ht

Output MA=CM CM=HT.CM AC=HT.ACC ME=HT.ME
