# Supplementary material for: Using structural equation modeling to detect response shifts and true change in discrete variables: an application to the items of the SF-36
Source: Qual Life Res. 2015 Dec 22;25:1361–83. doi: 10.1007/s11136-015-1195-0 (PMC4870306; doi:10.1007/s11136-015-1195-0)
Supplement: Supplementary file 3 — Supplementary material 3 (DOCX 32 kb) [file 11136_2015_1195_MOESM3_ESM.docx]

APPENDIX B

STAGE 2: LISREL SYNTAXES FOR ANALYSES OF ALL SUBSCALES OF THE SF-36

###########################################################################

# Mental Health

###########################################################################

STEP 1 : MEASUREMENT MODEL

da ng=1 ni=10 no=437 ma=cm

cm fi=MH.CM RE

me fi=MH.ME RE

wm fi=MH.ACC RE

LA

m1mh1 m1mh2 m1mh3 m1mh4 m1mh5

m2mh1 m2mh2 m2mh3 m2mh4 m2mh5

mo ny=10 ne=2 ly=fu,fr ps=sy,fr te=sy,fr al=fu,fi ty=fu,fr

LE

MH1 MH2

ma ps

1

0 1

pa ps

0

1 0

pa ly

1 0

1 0

1 0

1 0

1 0

0 1

0 1

0 1

0 1

0 1

pa te

1

0 1

0 0 1

0 0 0 1

0 0 0 0 1

1 0 0 0 0 1

0 1 0 0 0 0 1

0 0 1 0 0 0 0 1

0 0 0 1 0 0 0 0 1

0 0 0 0 1 0 0 0 0 1

fr te 5 3 te 10 8 te 10 3 te 8 5

fr te 5 4 te 10 9 te 10 4 te 9 5

ou dwls mi

STEP 2 : NO RESPONSE SHIFT MODEL

da ng=1 ni=10 no=437 ma=cm

cm fi=MH.CM RE

me fi=MH.ME RE

wm fi=MH.ACC RE

LA

m1mh1 m1mh2 m1mh3 m1mh4 m1mh5

m2mh1 m2mh2 m2mh3 m2mh4 m2mh5

mo ny=10 ne=2 ly=fu,fr ps=sy,fr te=sy,fr al=fu,fr ty=fu,fr

LE

MH1 MH2

ma ps

1

0 0

pa ps

0

1 1

fi al 1

va 0 al 1

pa ly

1 0

1 0

1 0

1 0

1 0

0 1

0 1

0 1

0 1

0 1

eq ly 1 1 ly 6 2

eq ly 2 1 ly 7 2

eq ly 3 1 ly 8 2

eq ly 4 1 ly 9 2

eq ly 5 1 ly 10 2

eq ty 1 ty 6

eq ty 2 ty 7

eq ty 3 ty 8

eq ty 4 ty 9

eq ty 5 ty 10

pa te

1

0 1

0 0 1

0 0 0 1

0 0 0 0 1

1 0 0 0 0 1

0 1 0 0 0 0 1

0 0 1 0 0 0 0 1

0 0 0 1 0 0 0 0 1

0 0 0 0 1 0 0 0 0 1

fr te 5 3 te 10 8 te 10 3 te 8 5

ou dwls mi so

STEP 3 : RESPONSE SHIFT MODEL

da ng=1 ni=10 no=437 ma=cm

cm fi=MH.CM RE

me fi=MH.ME RE

wm fi=MH.ACC RE

LA

m1mh1 m1mh2 m1mh3 m1mh4 m1mh5

m2mh1 m2mh2 m2mh3 m2mh4 m2mh5

mo ny=10 ne=2 ly=fu,fr ps=sy,fr te=sy,fr al=fu,fr ty=fu,fr

LE

MH1 MH2

ma ps

1

0 0

pa ps

0

1 1

fi al 1

va 0 al 1

pa ly

1 0

1 0

1 0

1 0

1 0

0 1

0 1

0 1

0 1

0 1

! Reprioritization RS

!eq ly 1 1 ly 6 2

eq ly 2 1 ly 7 2

eq ly 3 1 ly 8 2

eq ly 4 1 ly 9 2

eq ly 5 1 ly 10 2

! Recalibration RS

!eq ty 1 ty 6

eq ty 2 ty 7

eq ty 3 ty 8

eq ty 4 ty 9

! Recalibration RS

!eq ty 5 ty 10

pa te

1

0 1

0 0 1

0 0 0 1

0 0 0 0 1

1 0 0 0 0 1

0 1 0 0 0 0 1

0 0 1 0 0 0 0 1

0 0 0 1 0 0 0 0 1

0 0 0 0 1 0 0 0 0 1

fr te 5 3 te 10 8 te 10 3 te 8 5

ou dwls mi so

###########################################################################

# General Physical Health

###########################################################################

STEP 1 :MEASUREMENT MODEL

da ng=1 ni=10 no=437 ma=cm

cm fi=GH.CM RE

me fi=GH.ME RE

wm fi=GH.ACC RE

LA

m1gh1 m1gh2 m1gh3 m1gh4 m1gh5

m2gh1 m2gh2 m2gh3 m2gh4 m2gh5

mo ny= 10 ne=2 ly=fu,fr ps=sy,fr te=sy,fr al=fu,fi ty=fu,fr

LE

GH1 GH2

ma ps

1

0 1

pa ps

0

1 0

pa ly

1 0

1 0

1 0

1 0

1 0

0 1

0 1

0 1

0 1

0 1

pa te

1

0 1

0 0 1

0 0 0 1

0 0 0 0 1

1 0 0 0 0 1

0 1 0 0 0 0 1

0 0 1 0 0 0 0 1

0 0 0 1 0 0 0 0 1

0 0 0 0 1 0 0 0 0 1

ou dwls mi

STEP 2 : NO RESPONSE SHIFT MODEL

da ng=1 ni=10 no=437 ma=cm

cm fi=GH.CM RE

me fi=GH.ME RE

wm fi=GH.ACC RE

LA

m1gh1 m1gh2 m1gh3 m1gh4 m1gh5

m2gh1 m2gh2 m2gh3 m2gh4 m2gh5

mo ny= 10 ne=2 ly=fu,fr ps=sy,fr te=sy,fr al=fu,fr ty=fu,fr

LE

GH1 GH2

ma ps

1

0 0

pa ps

0

1 1

fi al 1

va 0 al 1

pa ly

1 0

1 0

1 0

1 0

1 0

0 1

0 1

0 1

0 1

0 1

eq ly 1 1 ly 6 2

eq ly 2 1 ly 7 2

eq ly 3 1 ly 8 2

eq ly 4 1 ly 9 2

eq ly 5 1 ly 10 2

eq ty 1 ty 6

eq ty 2 ty 7

eq ty 3 ty 8

eq ty 4 ty 9

eq ty 5 ty 10

pa te

1

0 1

0 0 1

0 0 0 1

0 0 0 0 1

1 0 0 0 0 1

0 1 0 0 0 0 1

0 0 1 0 0 0 0 1

0 0 0 1 0 0 0 0 1

0 0 0 0 1 0 0 0 0 1

ou dwls so mi

###########################################################################

# Physical Functioning

###########################################################################

STEP 1 : MEASUREMENT MODEL

da ng=1 ni=20 no=437 ma=cm

cm fi=PF.CM RE

me fi=PF.ME RE

wm fi=PF.ACC RE

LA

m1pf01 m1pf02 m1pf03 m1pf04 m1pf05 m1pf06 m1pf07 m1pf08 m1pf09 m1pf10

m2pf01 m2pf02 m2pf03 m2pf04 m2pf05 m2pf06 m2pf07 m2pf08 m2pf09 m2pf10

mo ny=20 ne=2 ly=fu,fr ps=sy,fr te=sy,fr al=fu,fi ty=fu,fr

LE

PF1 PF2

ma ps

1

0 1

pa ps

0

1 0

pa ly

1 0

1 0

1 0

1 0

1 0

1 0

1 0

1 0

1 0

1 0

0 1

0 1

0 1

0 1

0 1

0 1

0 1

0 1

0 1

0 1

pa te

1

0 1

0 0 1

0 0 0 1

0 0 0 0 1

0 0 0 0 0 1

0 0 0 0 0 0 1

0 0 0 0 0 0 0 1

0 0 0 0 0 0 0 0 1

0 0 0 0 0 0 0 0 0 1

1 0 0 0 0 0 0 0 0 0 1

0 1 0 0 0 0 0 0 0 0 0 1

0 0 1 0 0 0 0 0 0 0 0 0 1

0 0 0 1 0 0 0 0 0 0 0 0 0 1

0 0 0 0 1 0 0 0 0 0 0 0 0 0 1

0 0 0 0 0 1 0 0 0 0 0 0 0 0 0 1

0 0 0 0 0 0 1 0 0 0 0 0 0 0 0 0 1

0 0 0 0 0 0 0 1 0 0 0 0 0 0 0 0 0 1

0 0 0 0 0 0 0 0 1 0 0 0 0 0 0 0 0 0 1

0 0 0 0 0 0 0 0 0 1 0 0 0 0 0 0 0 0 0 1

fr te 3 2 te 13 12 te 13 2 te 12 3

fr te 5 4 te 15 14 te 15 4 te 14 5

ou dwls mi

STEP 2 : NO RESPONSE SHIFT MODEL

da ng=1 ni=20 no=437 ma=cm

cm fi=PF.CM RE

me fi=PF.ME RE

wm fi=PF.ACC RE

LA

m1pf01 m1pf02 m1pf03 m1pf04 m1pf05 m1pf06 m1pf07 m1pf08 m1pf09 m1pf10

m2pf01 m2pf02 m2pf03 m2pf04 m2pf05 m2pf06 m2pf07 m2pf08 m2pf09 m2pf10

mo ny=20 ne=2 ly=fu,fr ps=sy,fr te=sy,fr al=fu,fr ty=fu,fr

LE

PF1 PF2

ma ps

1

0 0

pa ps

0

1 1

fi al 1

va 0 al 1

pa ly

1 0

1 0

1 0

1 0

1 0

1 0

1 0

1 0

1 0

1 0

0 1

0 1

0 1

0 1

0 1

0 1

0 1

0 1

0 1

0 1

eq ly 1 1 ly 11 2

eq ly 2 1 ly 12 2

eq ly 3 1 ly 13 2

eq ly 4 1 ly 14 2

eq ly 5 1 ly 15 2

eq ly 6 1 ly 16 2

eq ly 7 1 ly 17 2

eq ly 8 1 ly 18 2

eq ly 9 1 ly 19 2

eq ly 10 1 ly 20 2

eq ty 1 ty 11

eq ty 2 ty 12

eq ty 3 ty 13

eq ty 4 ty 14

eq ty 5 ty 15

eq ty 6 ty 16

eq ty 7 ty 17

eq ty 8 ty 18

eq ty 9 ty 19

eq ty 10 ty 20

pa te

1

0 1

0 0 1

0 0 0 1

0 0 0 0 1

0 0 0 0 0 1

0 0 0 0 0 0 1

0 0 0 0 0 0 0 1

0 0 0 0 0 0 0 0 1

0 0 0 0 0 0 0 0 0 1

1 0 0 0 0 0 0 0 0 0 1

0 1 0 0 0 0 0 0 0 0 0 1

0 0 1 0 0 0 0 0 0 0 0 0 1

0 0 0 1 0 0 0 0 0 0 0 0 0 1

0 0 0 0 1 0 0 0 0 0 0 0 0 0 1

0 0 0 0 0 1 0 0 0 0 0 0 0 0 0 1

0 0 0 0 0 0 1 0 0 0 0 0 0 0 0 0 1

0 0 0 0 0 0 0 1 0 0 0 0 0 0 0 0 0 1

0 0 0 0 0 0 0 0 1 0 0 0 0 0 0 0 0 0 1

0 0 0 0 0 0 0 0 0 1 0 0 0 0 0 0 0 0 0 1

fr te 3 2 te 13 12 te 13 2 te 12 3

fr te 5 4 te 15 14 te 15 4 te 14 5

ou dwls mi so

STEP 3 : RESPONSE SHIFT MODEL

da ng=1 ni=20 no=437 ma=cm

cm fi=PF.CM RE

me fi=PF.ME RE

wm fi=PF.ACC RE

LA

m1pf01 m1pf02 m1pf03 m1pf04 m1pf05 m1pf06 m1pf07 m1pf08 m1pf09 m1pf10

m2pf01 m2pf02 m2pf03 m2pf04 m2pf05 m2pf06 m2pf07 m2pf08 m2pf09 m2pf10

mo ny=20 ne=2 ly=fu,fr ps=sy,fr te=sy,fr al=fu,fr ty=fu,fr

LE

PF1 PF2

ma ps

1

0 0

pa ps

0

1 1

fi al 1

va 0 al 1

pa ly

1 0

1 0

1 0

1 0

1 0

1 0

1 0

1 0

1 0

1 0

0 1

0 1

0 1

0 1

0 1

0 1

0 1

0 1

0 1

0 1

! Reprioritization RS

!eq ly 1 1 ly 11 2

eq ly 2 1 ly 12 2

eq ly 3 1 ly 13 2

eq ly 4 1 ly 14 2

eq ly 5 1 ly 15 2

eq ly 6 1 ly 16 2

eq ly 7 1 ly 17 2

eq ly 8 1 ly 18 2

eq ly 9 1 ly 19 2

! Reprioritization RS

!eq ly 10 1 ly 20 2

eq ty 1 ty 11

eq ty 2 ty 12

eq ty 3 ty 13

eq ty 4 ty 14

eq ty 5 ty 15

eq ty 6 ty 16

eq ty 7 ty 17

eq ty 8 ty 18

eq ty 9 ty 19

! Recallibration RS

!eq ty 10 ty 20

pa te

1

0 1

0 0 1

0 0 0 1

0 0 0 0 1

0 0 0 0 0 1

0 0 0 0 0 0 1

0 0 0 0 0 0 0 1

0 0 0 0 0 0 0 0 1

0 0 0 0 0 0 0 0 0 1

1 0 0 0 0 0 0 0 0 0 1

0 1 0 0 0 0 0 0 0 0 0 1

0 0 1 0 0 0 0 0 0 0 0 0 1

0 0 0 1 0 0 0 0 0 0 0 0 0 1

0 0 0 0 1 0 0 0 0 0 0 0 0 0 1

0 0 0 0 0 1 0 0 0 0 0 0 0 0 0 1

0 0 0 0 0 0 1 0 0 0 0 0 0 0 0 0 1

0 0 0 0 0 0 0 1 0 0 0 0 0 0 0 0 0 1

0 0 0 0 0 0 0 0 1 0 0 0 0 0 0 0 0 0 1

0 0 0 0 0 0 0 0 0 1 0 0 0 0 0 0 0 0 0 1

fr te 3 2 te 13 12 te 13 2 te 12 3

fr te 5 4 te 15 14 te 15 4 te 14 5

ou dwls mi so

###########################################################################

# Role Limitations due to Physical Health

###########################################################################

STEP 1 : MEASUREMENT MODEL

da ng=1 ni=8 no=437 ma=km

cm fi=RP.KM

me fi=RP.ME RE

wm fi=RP.ACC RE

LA

m1rp1 m1rp2 m1rp3 m1rp4

m2rp1 m2rp2 m2rp3 m2rp4

mo ny=8 ne=2 ly=fu,fr ps=sy,fr te=sy,fr al=fu,fi ty=fu,fr

LE

RP1 RP2

ma ps

1

0 1

pa ps

0

1 0

pa ly

1 0

1 0

1 0

1 0

0 1

0 1

0 1

0 1

pa te

1

0 1

0 0 1

0 0 0 1

1 0 0 0 1

0 1 0 0 0 1

0 0 1 0 0 0 1

0 0 0 1 0 0 0 1

ou dwls mi

STEP 2 :NO RESPONSE SHIFT MODEL

da ng=1 ni=8 no=437 ma=km

cm fi=RP.KM

me fi=RP.ME RE

wm fi=RP.ACC RE

LA

m1rp1 m1rp2 m1rp3 m1rp4

m2rp1 m2rp2 m2rp3 m2rp4

mo ny=8 ne=8 nk=2 ly=di,fi, ga=fu,fr ph=sy ps=sy,fr ty=ze al=fu,fr ka=fu,fi te=ze

ma ly

1 1 1 1 1 1 1 1

pa ly

0 0 0 0 1 1 1 1

pa ph

0

1 0

ma ph

1

0.5 1

pa ga

1 0

1 0

1 0

1 0

0 1

0 1

0 1

0 1

ma ga

0.5 0

0.5 0

0.5 0

0.5 0

0 0.5

0 0.5

0 0.5

0 0.5

eq ga 1 1 ga 5 2

eq ga 2 1 ga 6 2

eq ga 3 1 ga 7 2

eq ga 4 1 ga 8 2

pa ps

1

0 1

0 0 1

0 0 0 1

1 0 0 0 1

0 1 0 0 0 1

0 0 1 0 0 0 1

0 0 0 1 0 0 0 1

ma ps

.5

0 .5

0 0 .5

0 0 0 .5

.1 0 0 0 .5

0 .1 0 0 0 .5

0 0 .1 0 0 0 .5

0 0 0 .1 0 0 0 .5

pa al

1 1 1 1 1 1 1 1

ma al

0 0 0 0 0 0 0 0

eq al 1 al 5

eq al 2 al 6

eq al 3 al 7

eq al 4 al 8

pa ka

0 1

ma ka

0 0

ou dwls mi ns

STEP 3 : RESPONSE SHIFT MODEL

da ng=1 ni=8 no=437 ma=km

cm fi=RP.KM

me fi=RP.ME RE

wm fi=RP.ACC RE

LA

m1rp1 m1rp2 m1rp3 m1rp4

m2rp1 m2rp2 m2rp3 m2rp4

mo ny=8 ne=8 nk=2 ly=di,fi, ga=fu,fr ph=sy ps=sy,fr ty=ze al=fu,fr ka=fu,fi te=ze

ma ly

1 1 1 1 1 1 1 1

pa ly

0 0 0 0 1 1 1 1

pa ph

0

1 0

ma ph

1

0.5 1

pa ga

1 0

1 0

1 0

1 0

0 1

0 1

0 1

0 1

ma ga

0.5 0

0.5 0

0.5 0

0.5 0

0 0.5

0 0.5

0 0.5

0 0.5

eq ga 1 1 ga 5 2

eq ga 2 1 ga 6 2

eq ga 3 1 ga 7 2

eq ga 4 1 ga 8 2

pa ps

1

0 1

0 0 1

0 0 0 1

1 0 0 0 1

0 1 0 0 0 1

0 0 1 0 0 0 1

0 0 0 1 0 0 0 1

ma ps

.5

0 .5

0 0 .5

0 0 0 .5

.1 0 0 0 .5

0 .1 0 0 0 .5

0 0 .1 0 0 0 .5

0 0 0 .1 0 0 0 .5

pa al

1 1 1 1 1 1 1 1

ma al

0 0 0 0 0 0 0 0

!Recalibration RS

! eq al 1 al 5

eq al 2 al 6

eq al 3 al 7

eq al 4 al 8

pa ka

0 1

ma ka

0 0

ou dwls ns

###########################################################################

# Bodily Pain

###########################################################################

STEP 1 : MEASUREMENT MODEL

da ng=1 ni=4 no=437 ma=cm

cm fi=BP.CM RE

me fi=BP.ME RE

wm fi=BP.ACC RE

LA

m1bp1 m1bp2

m2bp1 m2bp2

mo ny=4 ne=2 ly=fu,fr ps=sy,fr te=sy,fr al=fu,fr ty-=fu,fr

LE

BP1 BP2

ma ps

1

0 1

pa ps

0

1 0

fi al 1 al 2

va 0 al 1 al 2

pa ly

1 0

1 0

0 1

0 1

pa te

1

0 1

0 0 1

0 0 0 1

ou dwls so

STEP 2 : NO RESPONSE SHIFT MODEL

da ng=1 ni=4 no=437 ma=cm

cm fi=BP.CM

me fi=BP.ME

wm fi=BP.ACC

LA

m1bp1 m1bp2

m2bp1 m2bp2

mo ny=4 ne=2 ly=fu,fr ps=sy,fr te=sy,fr al=fu,fr ty-=fu,fr

LE

BP1 BP2

ma ps

1

0 0

pa ps

0

1 1

fi al 1

va 0 al 1

pa ly

1 0

1 0

0 1

0 1

eq ly 1 1 ly 3 2

eq ly 2 1 ly 4 2

eq ty 1 ty 3

eq ty 2 ty 4

pa te

1

0 1

0 0 1

0 0 0 1

ou dwls mi so

STEP 3 : RESPONSE SHIFT MODEL

da ng=1 ni=4 no=437 ma=cm

cm fi=BP.CM

me fi=BP.ME

wm fi=BP.ACC

LA

m1bp1 m1bp2

m2bp1 m2bp2

mo ny=4 ne=2 ly=fu,fr ps=sy,fr te=sy,fr al=fu,fr ty-=fu,fr

LE

BP1 BP2

ma ps

1

0 0

pa ps

0

1 1

fi al 1

va 0 al 1

pa ly

1 0

1 0

0 1

0 1

eq ly 1 1 ly 3 2

eq ly 2 1 ly 4 2

! Recalibration RS

!eq ty 1 ty 3

eq ty 2 ty 4

pa te

1

0 1

0 0 1

0 0 0 1

ou dwls so

###########################################################################

# Social Functioning

###########################################################################

STEP 1 : MEASUREMENT MODEL

da ng=1 ni=4 no=437 ma=cm

cm fi=SF.CM RE

me fi=SF.ME RE

wm fi=SF.ACC RE

LA

m1sf1 m1sf2

m2sf1 m2sf2

mo ny=4 ne=2 ly=fu,fr ps=sy,fr te=sy,fr al=fu,fr ty-=fu,fr

LE

SF1 SF2

ma ps

1

0 1

pa ps

0

1 0

fi al 1 al 2

va 0 al 1 al 2

pa ly

1 0

1 0

0 1

0 1

eq ly 1 1 ly 2 1

eq ly 3 2 ly 4 2

pa te

1

0 1

1 0 1

0 1 0 1

ou dwls so

STEP 2 : NO RESPONSE SHIFT MODEL

da ng=1 ni=4 no=437 ma=cm

cm fi=SF.CM

me fi=SF.ME

wm fi=SF.ACC

LA

m1sf1 m1sf2

m2sf1 m2sf2

mo ny=4 ne=2 ly=fu,fr ps=sy,fr te=sy,fr al=fu,fr ty-=fu,fr

LE

SF1 SF2

ma ps

1

0 0

pa ps

0

1 1

fi al 1

va 0 al 1

pa ly

1 0

1 0

0 1

0 1

eq ly 1 1 ly 3 2 ly 2 1 ly 4 2

eq ty 1 ty 3

eq ty 2 ty 4

pa te

1

0 1

1 0 1

0 1 0 1

ou dwls so

###########################################################################

# Role Limitations due to Emotional Problems

###########################################################################

STEP 1 :MEASUREMENT MODEL

da ng=1 ni=6 no=437 ma=km

cm fi=RE.KM

me fi=RE.ME RE

wm fi=RE.ACC RE

LA

m1re1 m1re2 m1re3

m2re1 m2re2 m2re3

mo ny= 6 ne=2 ly=fu,fr ps=sy,fr te=sy,fr al=fu,fi ty=fu,fr

LE

RE1 RE2

pa ps

0

1 0

ma ps

1

0 1

pa ly

1 0

1 0

1 0

0 1

0 1

0 1

pa te

1

0 1

0 0 1

1 0 0 1

0 1 0 0 1

0 0 1 0 0 1

ou dwls mi

STEP 2 :NO RESPONSE SHIFT MODEL

da ng=1 ni=6 no=437 ma=km

cm fi=RE.KM

me fi=RE.ME RE

wm fi=RE.ACC RE

LA

m1re1 m1re2 m1re3

m2re1 m2re2 m2re3

mo ny=6 ne=6 nk=2 ly=di,fi, ga=fu,fr ph=sy ps=sy,fr ty=ze al=fu,fr ka=fu,fi te=ze

ma ly

1 1 1 1 1 1

pa ly

0 0 0 1 1 1

pa ph

0

1 0

ma ph

1

0.5 1

pa ga

1 0

1 0

1 0

0 1

0 1

0 1

ma ga

0.5 0

0.5 0

0.5 0

0 0.5

0 0.5

0 0.5

eq ga 1 1 ga 4 2

eq ga 2 1 ga 5 2

eq ga 3 1 ga 6 2

pa ps

1

0 1

0 0 1

1 0 0 1

0 1 0 0 1

0 0 1 0 0 1

ma ps

.5

0 .5

0 0 .5

.1 0 0 .5

0 .1 0 0 .5

0 0 .1 0 0 .5

pa al

1 1 1 1 1 1

ma al

0 0 0 0 0 0

eq al 1 al 4

eq al 2 al 5

eq al 3 al 6

pa ka

0 1

ma ka

0 0

ou dwls mi ns

###########################################################################

# Vitality

###########################################################################

STEP 1 : MEASUREMENT MODEL

da ng=1 ni=8 no=437 ma=cm

cm fi=VT.CM RE

me fi=VT.ME RE

wm fi=VT.ACC RE

LA

m1vt1 m1vt2 m1vt3 m1vt4

m2vt1 m2vt2 m2vt3 m2vt4

mo ny=8 ne=2 ly=fu,fr ps=sy,fr te=sy,fr al=fu,fi ty=fu,fr

LE

VT1 VT2

ma ps

1

0 1

pa ps

0

1 0

pa ly

1 0

1 0

1 0

1 0

0 1

0 1

0 1

0 1

pa te

1

0 1

0 0 1

0 0 0 1

1 0 0 0 1

0 1 0 0 0 1

0 0 1 0 0 0 1

0 0 0 1 0 0 0 1

fr te 4 3 te 8 7 te 8 3 te 7 4

ou dwls mi

STEP 2 : NO RESPONSE SHIFT MODEL

da ng=1 ni=8 no=437 ma=cm

cm fi=VT.CM RE

me fi=VT.ME RE

wm fi=VT.ACC RE

LA

m1vt1 m1vt2 m1vt3 m1vt4

m2vt1 m2vt2 m2vt3 m2vt4

mo ny=8 ne=2 ly=fu,fr ps=sy,fr te=sy,fr al=fu,fr ty=fu,fr

LE

VT1 VT2

ma ps

1

0 0

pa ps

0

1 1

fi al 1

va 0 al 1

pa ly

1 0

1 0

1 0

1 0

0 1

0 1

0 1

0 1

eq ly 1 1 ly 5 2

eq ly 2 1 ly 6 2

eq ly 3 1 ly 7 2

eq ly 4 1 ly 8 2

eq ty 1 ty 5

eq ty 2 ty 6

eq ty 3 ty 7

eq ty 4 ty 8

pa te

1

0 1

0 0 1

0 0 0 1

1 0 0 0 1

0 1 0 0 0 1

0 0 1 0 0 0 1

0 0 0 1 0 0 0 1

fr te 4 3 te 8 7 te 8 3 te 7 4

ou dwls so mi

###########################################################################

# Health Comparison

###########################################################################

NA
