# Supplementary material for: Using structural equation modeling to detect response shifts and true change in discrete variables: an application to the items of the SF-36
Source: Qual Life Res. 2015 Dec 22;25:1361–83. doi: 10.1007/s11136-015-1195-0 (PMC4870306; doi:10.1007/s11136-015-1195-0)
Supplement: Supplementary file 4 — Supplementary material 4 (DOCX 22 kb) [file 11136_2015_1195_MOESM4_ESM.docx]

APPENDIX C

SYNTAXES FOR CALCULATION OF APPROXIMATE FIT INDICES AND DIFFERENCE IN FIT

### calculate confidence interval ncp given chi-square value

ncp.conf=function (chival, df, conf, prec = 1e-05)

{

result <- NA

ulim <- 1 - (1 - conf)/2

lc <- c(0.001, chival/2, chival)

while (pchisq(chival, df, lc[1]) < ulim) {

if (pchisq(chival, df) < ulim) {

result <- (c(0, pchisq(chival, df)))

break

}

lc <- c(lc[1]/4, lc[1], lc[3])

}

diff <- 1

if (all(is.na(result))) {

while (diff > prec) {

if (pchisq(chival, df, lc[2]) < ulim)

lc <- c(lc[1], (lc[1] + lc[2])/2, lc[2])

else lc <- c(lc[2], (lc[2] + lc[3])/2, lc[3])

diff <- abs(pchisq(chival, df, lc[2]) - ulim)

ucdf <- pchisq(chival, df, lc[2])

}

result <- c(lc[2], ucdf)

}

uc <- c(chival, 2 * chival, 3 * chival)

llim <- (1 - conf)/2

while (pchisq(chival, df, uc[1]) < llim && uc > prec) {

uc <- c(uc[1]/4, uc[1], uc[3])

}

while (pchisq(chival, df, uc[3]) > llim) {

uc <- c(uc[1], uc[3], uc[3] + chival)

}

diff <- 1

count <- 0

while (diff > prec) {

if (pchisq(chival, df, uc[2]) < llim)

uc <- c(uc[1], (uc[1] + uc[2])/2, uc[2])

else uc <- c(uc[2], (uc[2] + uc[3])/2, uc[3])

diff <- abs(pchisq(chival, df, uc[2]) - llim)

lcdf <- pchisq(chival, df, uc[2])

count <- count + 1

if (count > 1000) {

warning("Convergence not reached in chi.noncentral.conf .")

uc[2] <- Inf

lcdf <- 0

break

}

}

result <- rbind(result, c(uc[2], lcdf))

rownames(result) <- c("Lower", "Upper")

colnames(result) <- c("Non-Central", "%")

result

}

### calculate approximate fit & difference in fit

FIT = function(chi1,df1,Ntotal,nvar,chi2=NULL,df2=NULL,conf=.90,ngroups=1,RMSEA0=.05){

### parameters first model

pstar=(nvar*(nvar+1))/2

q1=pstar-df1

p1 = 1 - pchisq(chi1,df1)

N=Ntotal

### calculate confidence interval ncp for chi-square value first model

options(warn = -1)

ncp.conf.limits1 = ncp.conf(chival = chi1, df = df1, conf=conf)

lbound.ncp1 = ncp.conf.limits1[1]

if(is.na(lbound.ncp1)) lbound.ncp1=0

ubound.ncp1 = ncp.conf.limits1[2]

### calculate RMSEA first model

RMSEA1 = round(sqrt(ngroups)*sqrt(max(chi1-df1,0)/(df1 * (Ntotal - 1))),4)

RMSEA1.lbound = sqrt(ngroups)*sqrt(lbound.ncp1/(df1 * (Ntotal - 1)))

if (is.na(RMSEA1.lbound)) RMSEA1.lbound = 0

RMSEA1.ubound = sqrt(ngroups)*sqrt(ubound.ncp1/(df1 * (Ntotal - 1)))

RMSEA1.conf = paste(round(RMSEA1.lbound,4),"-",round(RMSEA1.ubound,4))

RMSEA1.closefit = 1-pchisq(chi1,df1,(RMSEA0*RMSEA0*df1*(Ntotal-1)))

RMSEA1.notclosefit = 1-RMSEA1.closefit

### calculate ECVI first model using formula Browne: F + 2*q/N-1

ECVI1 = (chi1/(N-ngroups)) + (2*(q1/(N-ngroups)))

ECVI1.lbound = ((lbound.ncp1+df1)/(N-ngroups)) + (2*(q1/(N-ngroups)))

if (is.na(ECVI1.lbound)) ECVI1.lbound = 0

ECVI1.ubound = ( (ubound.ncp1+df1)/(N-ngroups)) + (2*(q1/(N-ngroups)))

ECVI1.conf = paste(round(ECVI1.lbound,4),"-",round(ECVI1.ubound,4))

### result model 1

MODEL1.result = data.frame(CHISQ=chi1, DF=df1,

RMSEA = RMSEA1, RMSEA.conf.int = RMSEA1.conf, p.closefit = round(RMSEA1.closefit,3),

ECVI = round(ECVI1,4), ECVI.conf.int = ECVI1.conf)

### output

output = list( model1=MODEL1.result)

if(!is.null(chi2)){

### parameters second model

pstar=(nvar*(nvar+1))/2

q2=pstar-df2

p2 = 1 - pchisq(chi2,df2)

N=Ntotal

### parameters for difference between models

chi.diff = chi1-chi2

df.diff = df1-df2

q.diff = -df.diff

N.diff= Ntotal

p.diff = 1 - pchisq(chi.diff,df.diff)

### calculate confidence interval ncp associated with chisquare value second model

options(warn = -1)

ncp.conf.limits2 = ncp.conf(chival = chi2, df = df2, conf=conf)

lbound.ncp2 = ncp.conf.limits2[1]

if(is.na(lbound.ncp1)) lbound.ncp2=0

ubound.ncp2 = ncp.conf.limits2[2]

### calculate confidence interval ncp associated with difference in chisquare values

options(warn = -1)

ncp.conf.limits.diff = ncp.conf(chival = chi.diff, df = df.diff, conf=conf)

lbound.ncp.diff = ncp.conf.limits.diff[1]

if(is.na(lbound.ncp.diff)) lbound.ncp.diff=0

ubound.ncp.diff = ncp.conf.limits.diff[2]

### calculate RMSEA for second model

RMSEA2 = round(sqrt(ngroups)*sqrt(max(chi2-df2,0)/(df2 * (Ntotal - 1))),4)

RMSEA2.lbound = sqrt(ngroups)*sqrt(lbound.ncp2/(df2 * (Ntotal - 1)))

if (is.na(RMSEA2.lbound)) RMSEA2.lbound = 0

RMSEA2.ubound = sqrt(ngroups)*sqrt(ubound.ncp2/(df2 * (Ntotal - 1)))

RMSEA2.conf = paste(round(RMSEA2.lbound,4),"-",round(RMSEA2.ubound,4))

RMSEA2.closefit = 1-pchisq(chi2,df2,(RMSEA0*RMSEA0*df2*(Ntotal-1)))

RMSEA2.notclosefit = 1-RMSEA2.closefit

RMSEA2.result = data.frame(RMSEA = RMSEA2, confidence.int = RMSEA2.conf,

p.closefit = round(RMSEA2.closefit,3), p.notclosefit = round(RMSEA2.notclosefit,3))

### calculate ECVI second model with formula Browne: F + 2*q/N-1

ECVI2 = (chi2/(N-ngroups)) + (2*(q2/(N-ngroups)))

ECVI2.lbound = ((lbound.ncp2+df2)/(N-ngroups)) + (2*(q2/(N-ngroups)))

if (is.na(ECVI2.lbound)) ECVI2.lbound = 0

ECVI2.ubound = ( (ubound.ncp2+df2)/(N-ngroups)) + (2*(q2/(N-ngroups)))

ECVI2.conf = paste(round(ECVI2.lbound,4),"-",round(ECVI2.ubound,4))

### result model 2

MODEL2.result = data.frame(CHISQ=chi2, DF=df2,

RMSEA = RMSEA2, RMSEA.conf.int = RMSEA2.conf, p.closefit = round(RMSEA2.closefit,3),

ECVI = round(ECVI2,4), ECVI.conf.int = ECVI2.conf)

### calculate ECVI difference with difference in chi-square value. Browne: F + 2*q/N-1

ECVI.diff1 = round(( (chi.diff/(N.diff-1)) + (2*(q.diff/(N.diff-1))) ),4)

ECVI.lbound.diff1 = ((lbound.ncp.diff+df.diff)/(N.diff-1)) + (2*(q.diff/(N.diff-1)))

if (is.na(ECVI.lbound.diff1)) ECVI.lbound.diff1 = 0

ECVI.ubound.diff1 = ((ubound.ncp.diff+df.diff)/(N.diff-1)) + (2*(q.diff/(N.diff-1)))

ECVI.diff.conf1 = paste(round(ECVI.lbound.diff1,4),"-",round(ECVI.ubound.diff1,4))

ECVI.diff.result1 = paste(ECVI.diff1,",","[",round(ECVI.lbound.diff1,4),";",round(ECVI.ubound.diff1,4),"]")

### result difference

DIFFERENCE.result = data.frame(CHISQ.diff=chi.diff,DF.diff=df.diff,p.diff=round(p.diff,4),

ECVI.diff=round(ECVI.diff1,4), ECVI.conf.int=ECVI.diff.conf1)

### output

output = list( model1=MODEL1.result,

model2=MODEL2.result,

difference=DIFFERENCE.result)

}

return(output)

}

#### STAGE 1

#### TESTING EQUALITY THRESHOLDS

#mh1

FIT(chi1=33.494,df1=27,chi2=29.350,df2=24,Ntotal=437,nvar=21)

#mh2

FIT(chi1=31.133,df1=27,chi2=30.543,df2=24,Ntotal=437,nvar=21)

#mh3

FIT(chi1=65.603,df1=27,chi2=50.044,df2=24,Ntotal=437,nvar=21)

#mh4

FIT(chi1=75.064,df1=27,chi2=69.543,df2=24,Ntotal=437,nvar=21)

#mh5

FIT(chi1=60.653,df1=27,chi2=55.246,df2=24,Ntotal=437,nvar=21)

#gh1

FIT(chi1=32.979,df1=17,chi2=29.371,df2=15,Ntotal=437,nvar=10)

#gh2

FIT(chi1=72.150,df1=17,chi2=68.519,df2=15,Ntotal=437,nvar=10)

#gh3

FIT(chi1=54.792,df1=17,chi2=49.909,df2=15,Ntotal=437,nvar=12)

#gh4

FIT(chi1=39.588,df1=17,chi2=37.336,df2=15,Ntotal=437,nvar=12)

#gh5

FIT(chi1=86.734,df1=17,chi2=81.821,df2=15,Ntotal=437,nvar=12)

#sf1

FIT(chi1=17.573,df1=17,chi2=16.090,df2=15,Ntotal=437,nvar=10)

#sf2

FIT(chi1=34.044,df1=17,chi2=30.956,df2=15,Ntotal=437,nvar=10)

#bp1

FIT(chi1=30.955,df1=27,chi2=21.187,df2=24,Ntotal=437,nvar=12)

#bp2

FIT(chi1=23.280,df1=17,chi2=22.703,df2=15,Ntotal=437,nvar=12)

#vt1

FIT(chi1=81.908,df1=27,chi2=75.243,df2=24,Ntotal=437,nvar=8)

#vt2

FIT(chi1=55.516,df1=27,chi2=54.471,df2=24,Ntotal=437,nvar=8)

#vt3

FIT(chi1=44.146,df1=27,chi2=40.685,df2=24,Ntotal=437,nvar=8)

#vt4

FIT(chi1=55.331,df1=27,chi2=49.476,df2=24,Ntotal=437,nvar=8)

#ht

FIT(chi1=34.237,df1=17,chi2=27.280,df2=15,Ntotal=437,nvar=10)

#### STAGE 2

#### TESTING MEASUREMENT MODELS

#### TESTING DIFFERENCES NO-RS vs MM

#### ADDING RESPONSE SHIFT EFFECTS

#### TESTING DIFFERENCES RS vs MM

########################## MH

# MH Measurement Model

FIT(chi1=61.525,df1=25,Ntotal=437,nvar=10) # NNT overall model fit MM + residual 5-3

# -> close fit cannot be rejected

# MH No Response Shift Model

FIT(chi1=160.805,df1=33,Ntotal=437,nvar=8) # NNT overall model fit noRS model

FIT(chi1=160.805,df1=33,chi2=61.525,df2=25,Ntotal=437,nvar=10) # NNT difference noRS - MM

# -> signifcant difference chi-square and ECVI

# -> evidence response shift

# intercept item 24

FIT(chi1=106.487,df1=32,Ntotal=437,nvar=8) # NNT overall model fit noRS model + int item 24

# -> still no close fit

FIT(chi1=160.805,df1=33,chi2=106.487,df2=32,Ntotal=437,nvar=10) # NNT diff noRS+int - noRS

# -> significant improvement

FIT(chi1=106.487,df1=32,chi2=61.525,df2=25,Ntotal=437,nvar=10) # NNT diff noRS+int - MM

# -> still significant difference

# factor loading item 24

FIT(chi1=77.136,df1=31,Ntotal=437,nvar=8) # NNT overall model fit noRS model + int&lad item 24

# -> still no close fit

FIT(chi1=106.487,df1=32,chi2=77.136,df2=31,Ntotal=437,nvar=10) # NNT diff noRS+int

# -> significant improvement

FIT(chi1=77.136,df1=31,chi2=61.525,df2=25,Ntotal=437,nvar=10) # NNT diff noRS+int&lad - MM

# -> significant CHISQ but no longer significant difference (according to ECVI)

# intercept item 30

FIT(chi1=62.954,df1=30,Ntotal=437,nvar=8) # NNT noRS model + int&lad item 24 + int item 30

# -> now we have close fit

FIT(chi1=77.136,df1=32,chi2=62.954,df2=31,Ntotal=437,nvar=10) # NNT diff noRS

# -> significant improvement

FIT(chi1=62.954,df1=30,chi2=61.525,df2=25,Ntotal=437,nvar=10 # NNT difference MM

# -> no longer significant difference (according to both CHISQ and ECVI)

# MH Response Shift Model

FIT(chi1=62.954,df1=30,Ntotal=437,nvar=8) # NNT overall model fit RS model

########################## GH

# GH Measurement Model

FIT(chi1=61.286,df1=29,Ntotal=437,nvar=10) # NNT overall model fit MM

# -> close fit cannot be rejected

# GH No Response Shift Model

FIT(chi1=72.601,df1=37,Ntotal=437,nvar=10) # NNT overall model fit noRS

# -> still close fit

# Minimum Fit value: 116.524

FIT(chi1=72.601,df1=37,chi2=61.286,df2=29,Ntotal=437,nvar=8) # NNT difference with MM

# -> no evidence RS

########################## PF

# PF Measurement Model

FIT(chi1=339.055,df1=151,Ntotal=437,nvar=20) # SB overall model fit MM

# -> close fit cannot be rejected

# PF No Response Shift Model

FIT(chi1=477.640,df1=169,Ntotal=437,nvar=20) # SB overall model fit noRS

# -> no close fit

FIT(chi1=1718.731,df1=169,chi2=1338.077,df2=151,nvar=20,Ntotal=437) # C1 difference with MM

# -> difference in model fit -> RS

# loading item 12

FIT(chi1=448.699,df1=168,Ntotal=437,nvar=20) # SB overall model fit noRS + lad

# -> no close fit

FIT(chi1=1718.731,df1=169,chi2=1572.533,df2=168,nvar=20,Ntotal=437) # C1 difference with noRS

# -> significant improvement

FIT(chi1=1572.533,df1=168,chi2=1338.077,df2=151,nvar=20,Ntotal=437) # C1 difference with MM

# -> still significant difference in model fit

# intercept item 12

FIT(chi1=377.289,df1=167,Ntotal=437,nvar=20) # SB overall model fit noRS + lad&int

# -> close fit

FIT(chi1=1572.533,df1=168,chi2=1398.823,df2=167,nvar=20,Ntotal=437) # C1 diff with noRS+lad

# -> significant improvement

FIT(chi1=1398.823,df1=167,chi2=1338.077,df2=151,nvar=20,Ntotal=437) # C1 difference with MM

# -> still significant difference in model fit

# loading item 3

FIT(chi1=374.981,df1=166,Ntotal=437,nvar=20) # SB overall model fit noRS + lad&int + lad

# -> close fit

FIT(chi1=1718.731,df1=169,chi2=1384.830,df2=166,nvar=20,Ntotal=437) # C1 diff noRS+lad&int10

# -> significant improvement

FIT(chi1=1384.830,df1=166,chi2=1338.077,df2=151,nvar=20,Ntotal=437) # C1 difference with MM

# -> equivalent approximate fit

# PF Response Shift Model

FIT(chi1=374.981,df1=166,Ntotal=437,nvar=20) # SB overall model fit RS

########################## RP

# RP Measurement Model

FIT(chi1=29.727,df1=15,Ntotal=437,nvar=8) # NNT overall model fit MM

# -> close fit cannot be rejected

# RP No Response Shift Model

FIT(chi1=72.543,df1=18,Ntotal=437,nvar=8) # NNT overall model fit noRS

# -> close fit should be rejected

# -> evidence response shift

# intercept item 13

FIT(chi1=51.313,df1=17,Ntotal=437,nvar=8) # NNT overall model fit noRS + intercept item 13

# -> close fit cannot be rejected

FIT(chi1=72.543,df1=18,chi2=51.313,df2=17,Ntotal=437,nvar=8) # NNT difference noRS

# -> significant improvement

# -> accept model based on overall fit

########################## BP

# BP Measurement Model with residual covariances restricted to zero

FIT(chi1=1.798,df1=1,Ntotal=437,nvar=4) # NNT overall model fit MM

# BP No Response Shift Model with residual covariances restricted to zero

FIT(chi1=39.766,df1=3,Ntotal=437,nvar=4) # NNT overall model fit noRS

FIT(chi1=39.766,df1=3,chi2=1.798,df2=1,Ntotal=437,nvar=4) # difference in fit noRS-MM

# -> significant deterioration

# evidence RS

# BP Response Shift Model with residual covariances restricted to zero

FIT(chi1=5.941,df1=2,Ntotal=437,nvar=4) # NNT overall model fit RS

FIT(chi1=39.766,df1=3,chi2=5.941,df2=2,Ntotal=437,nvar=4) # difference in fit RS-noRS

# -> significant improvement

FIT(chi1=5.941,df1=2,chi2=1.798,df2=1,Ntotal=437,nvar=4) # difference in fit RS-MM

# -> no significant difference anymore

########################## SF

# SF Measurement Model with equal factor loadings

FIT(chi1=0.143,df1=1,Ntotal=437,nvar=4) # NNT overall model fit MM

# SF No Response Shift Model with equal factor loadings

FIT(chi1=1.303,df1=2,Ntotal=437,nvar=4) # NNT overall model fit noRS

FIT(chi1=1.303,df1=2,chi2=0.143,df2=1,Ntotal=437,nvar=4) # difference in fit noRS-MM

# -> not significant

# no RS

########################## RE

# RE Measurement Model

FIT(chi1=13.022,df1=5,Ntotal=437,nvar=6) # NNT overall model fit MM

# -> close fit cannot be rejected

# RE No Response Shift Model

FIT(chi1=17.834,df1=7,Ntotal=437,nvar=6) # NNT overall model fit noRS

# -> close fit cannot be rejected

# -> based on overall fit we accept no RS

########################## VT

# VT Measurement Model

FIT(chi1=4.730,df1=11,Ntotal=437,nvar=8) # NNT overall model fit MM + res 3-4

# -> close fit cannot be rejected

# VT No Response Shift Model

FIT(chi1=12.326,df1=17,Ntotal=437,nvar=8) # NNT overall model fit noRS

# -> close fit cannot be rejected

FIT(chi1=12.326,df1=17,chi2=4.730,df2=11,Ntotal=437,nvar=8) # NNT difference noRS - MM

# -> no significant difference

# no evidence RS
